# Improved prediction of gene expression through integrating cell signalling models with machine learning

**DOI:** 10.1186/s12859-022-04787-8

**Published:** 2022-08-06

**Authors:** Nada Al taweraqi, Ross D. King

**Affiliations:** 1 Department of Computer Science, University of Manchester, Manchester, UK; 2 Department of Computer Science, Taif University, Taif, Saudi Arabia; 3 Department of Chemical Engineering and Biotechnology, University of Cambridge, Cambridge, UK; 4 Department of Biology and Biological Engineering, Chalmers University of Technology, Gothenburg, Sweden; 5 Alan Turing Institute, London, UK

**Keywords:** Machine learning, Multi-target regression, Gene expression

## Abstract

**Background:**

A key problem in bioinformatics is that of predicting gene expression levels. There are two broad approaches: use of mechanistic models that aim to directly simulate the underlying biology, and use of machine learning (ML) to empirically predict expression levels from descriptors of the experiments. There are advantages and disadvantages to both approaches: mechanistic models more directly reflect the underlying biological causation, but do not directly utilize the available empirical data; while ML methods do not fully utilize existing biological knowledge.

**Results:**

Here, we investigate overcoming these disadvantages by integrating mechanistic cell signalling models with ML. Our approach to integration is to augment ML with similarity features (attributes) computed from cell signalling models. Seven sets of different similarity feature were generated using graph theory. Each set of features was in turn used to learn multi-target regression models. All the features have significantly improved accuracy over the baseline model - without the similarity features. Finally, the seven multi-target regression models were stacked together to form an overall prediction model that was significantly better than the baseline on 95% of genes on an independent test set. The similarity features enable this stacking model to provide interpretable knowledge about cancer, e.g. the role of ERBB3 in the MCF7 breast cancer cell line.

**Conclusion:**

Integrating mechanistic models as graphs helps to both improve the predictive results of machine learning models, and to provide biological knowledge about genes that can help in building state-of-the-art mechanistic models.

**Supplementary Information:**

The online version contains supplementary material available at 10.1186/s12859-022-04787-8.

## Background

Scientific understanding is demonstrated by making successful experimental predictions. The traditional approach to prediction in science is to build a model, mathematical or computational, that directly reflects the causation in the underlying physical system. For example, to predict gene expression levels one first builds a model of cell signalling and the control of gene expression that involves the genes/proteins known to involved, and then from initial conditions one uses the model to simulate the system to make predictions. In contrast, machine learning (ML) has been repeatedly shown to be able to make successful scientific predictions by learning input/output models. In the case of predicting gene expression levels the inputs are descriptions of the experimental conditions, and the output is gene expression levels. This ML approach does not necessarily reflect the underlying physical systems. However, in practice ML predictions are often more accurate than using models that directly reflect the underlying physical system. This is possible because many cases there is insufficient knowledge of the underlying system to form good mechanistic models, e.g. important structural elements or parameters may be missing or incorrect. It is also possible that computationally required approximations in the simulation result in inaccurate predictions.

When comparing traditional modelling and ML it is instructive to compare weather forecasting and protein structure predictions. The currently most successful approach to weather forecasting is to physically simulate weather systems [1], and machine learning for weather forecasting has been less successful [2]. The opposite situation occurs with protein structure prediction, where the currently most successful approach is to use machine learning, while simulation of protein folding has been less successful [3–5].

### Predicting gene expression levels

Predicting gene expression levels is a fundamental task in bioinformatics. There are two broad approaches: application of mechanistic models that aim to directly simulate the underlying biology, and use of machine learning (ML) to learn models that map cell input state to output expression levels. (Note that the term ‘model’ is used quite differently in these two approaches.) The two types of models, machine learning and mechanism-based, have their strengths and weaknesses: mechanistic models more directly reflect the underlying biology, but do not directly utilize the available empirical data; while ML methods do not fully utilize existing biological knowledge. One way to overcome these limitations might be to integrate both of them into a single framework. The simplest way to achieve this is use the mechanistic models to assist the ML models.

Mechanism-based cell signalling models can be used to directly predict gene expressions, as they describe the way different genes and proteins communicate and the results of their interactions. We define mechanistic models as in [6]: ‘an internal division of causal labour whereby different components perform different causal roles’. These models reflect the causal relationships between different genes and their interactions with drugs within pathways. The knowledge in such models can be divided into two parts: declarative (encyclopaedic), and procedural (simulation). While such mechanistic models have proven to be extremely useful, they are still, by definition, limited, and do not fully exploit the explosion of available biomedical data [7].

In designing a ML approach to predicting gene expression there are two broad choices to make: the type of learning method, and the type of bioinformatic information to use for the learning. Many machine learning methods have been used to predict variations in gene expression levels. Bayesian networks were used by Beer et al. [8], who utilised gene sequence information to predict a predefined expression pattern. These expression patterns were defined based on the k-means clustering algorithm. Predictions have also been made using deep learning, such as Chen et al. [9], who used a deep learning model with dropout as a regularization to infer the gene expression level for 21.000 target genes from a subset of genes that do not exceed 1000; Singh et al. [10], who developed a convolutional neural network (CNN) system called “DeepChrome”, predicts the gene expression levels using histone modifications as input.

When predicting gene expression levels we are typically interested in predicting the expression levels of many genes in parallel. This suggest the use of multi-task or transfer machine learning. Multi-task learning [11] is the branch of machine learning in which related problems (tasks) are learned simultaneously, with the aim of exploiting similarities between the problems to improve performance. The idea of transfer learning is similar, to utilise knowledge from one or more source domains, and reuse this knowledge in a target domain where data is scarce, with the aim of building better performing learning models in the target domain [12].

**Fig. 1:**
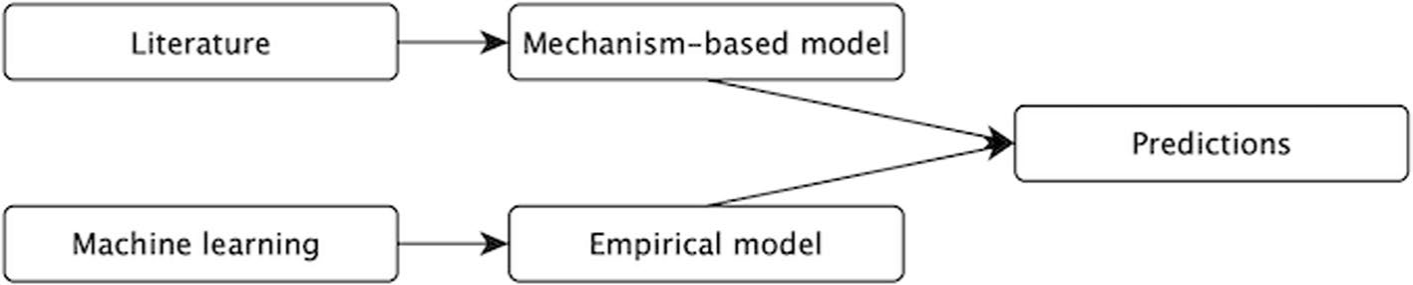
An overview of the general approach. We take advantage of graph analysis algorithms to convert knowledge from the literature to features that enhance the performance of multi-target machine learning models

This work aims to predict gene expression levels simultaneously, utilizing knowledge of their interactions taken from the literature. We report the results with and without this extra element to validate the influence of adding mechanism knowledge to the model. The baseline model is the model without this knowledge. The general approach of our research is illustrated in Fig. 1.

## Results

### Prediction results

**Fig. 2:**
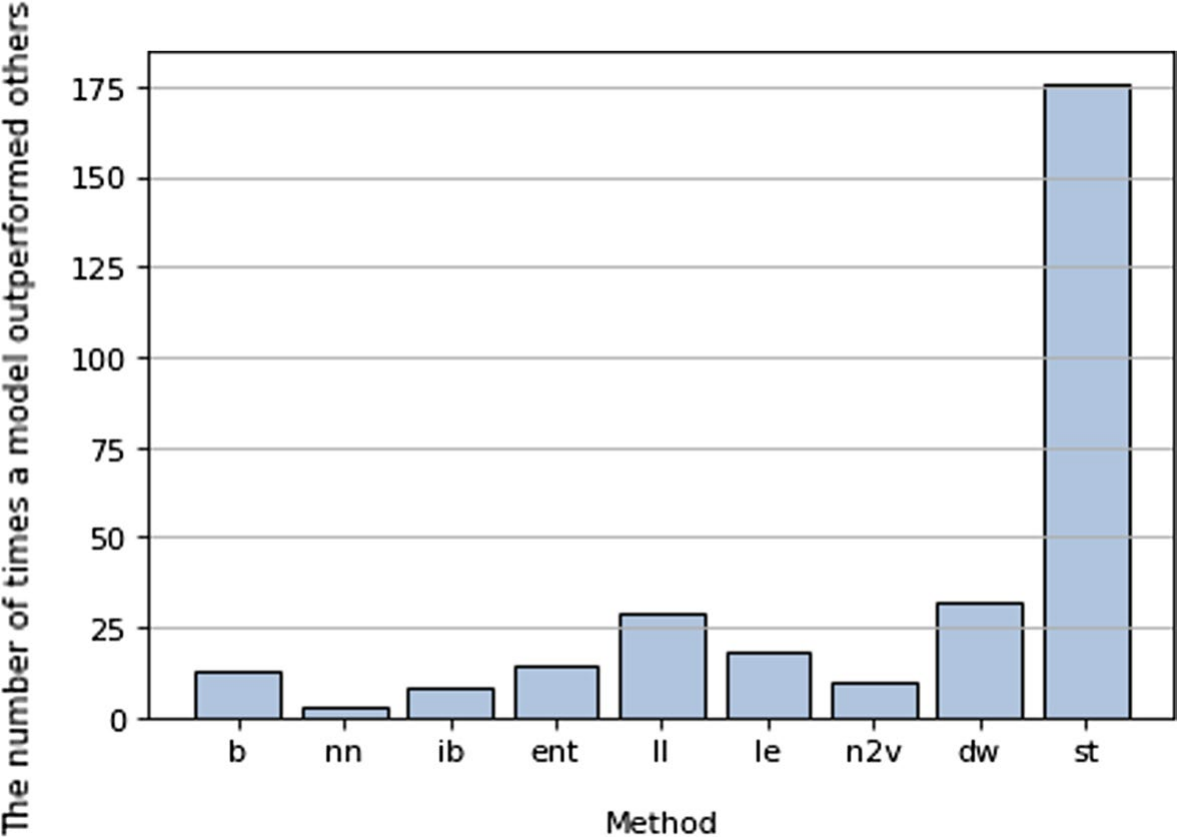
The results of models built based in different methods, b: baseline, nn: common neighbour, ib: local path index, ent: entropy-based method , ll: locally linear embedding, le: Laplacian eigenmaps, n2v: Nod2vec, dw: Deepwalk , st: stacking model. The figure shows the number of times a model outperformed other models for each gene, Stacking model surpassed other models by a wide margin

We compared (1) the baseline ML method, (2) the baseline method augmented with information from the seven graph structure methods, and (3) stacking all the methods together. The results of the first round of training are shown in Additional file 1: Table S1. In Additional file 1: Table S2 in the appendix, the results of the second round, where the accuracy of the models is measured by $R^2$. Figure 2 shows the performance of each model.

**Fig. 3:**
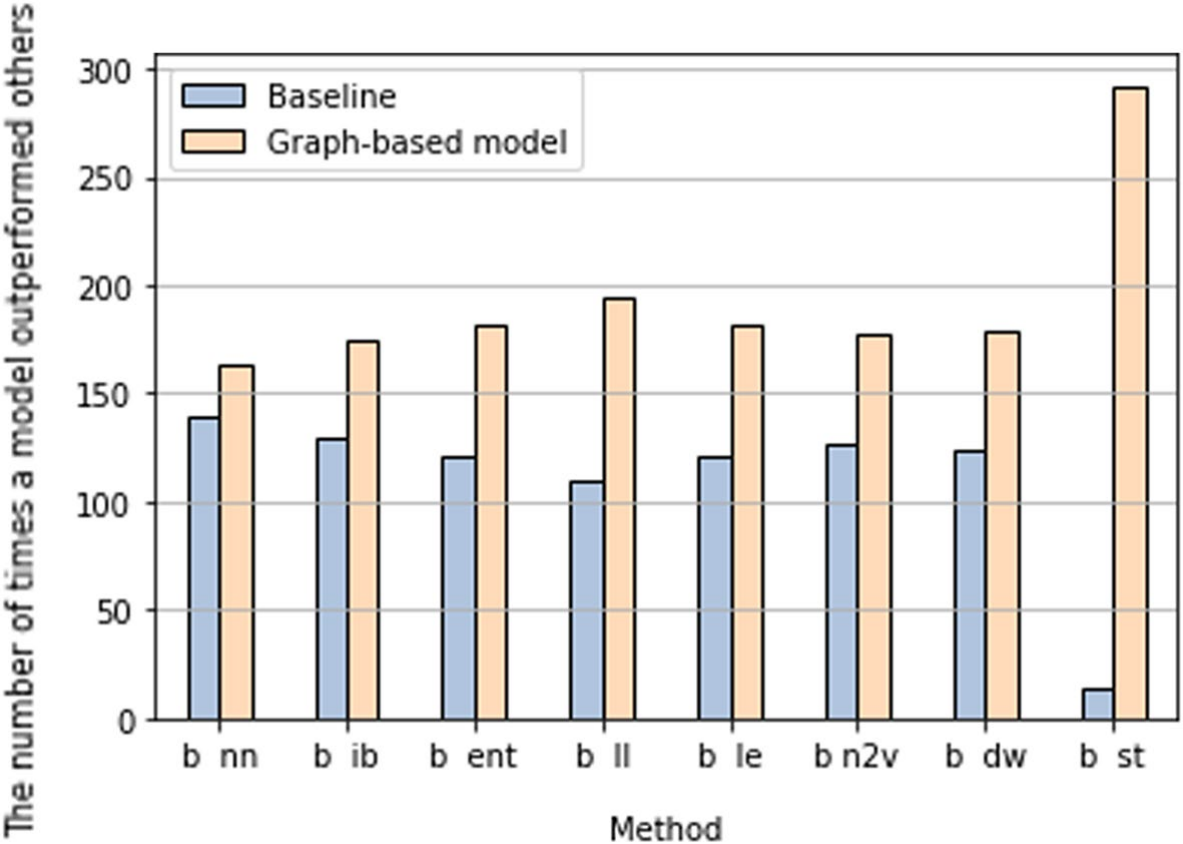
A pairwise comparison between baseline and models built based on graph methods, b: baseline, nn: common neighbour, ib: local path index, ent: entropy-based method , ll: locally linear embedding, le: Laplacian eigenmaps, n2v: Nod2vec, dw: Deepwalk , st: stacking model. The y axis shows the number of times a model outperformed the other. When compared to the base model, all graph-based models recorded higher scores

As it is a multi-target problem, where we build multiple models for equally significant targets, we measure the quality of the method based on the number of times the model scored the highest accuracy for each target. In this way, we can rank methods based on their accuracy, as in Fig. 2. Also, we directly compare each method with the baseline in Fig. 3, as we are looking at the possible improvement any of these methods can achieve.

For example, in Fig. 2, the entropy-based model registered the highest accuracy in the models for only 14 genes, compared to stacking- which is the best performing method- this is considered a poor result. However, it is still better than the local bath index method for example. Compared to the baseline as in Fig. 3, the entropy-based method noticeably outperforms the baseline model in more than 60 per cent of genes. With the null hypothesis that there is no difference in performance between the baseline and entropy-based models, the significance test resulted in the *p*-value of 3.7e−08, which is strong evidence against the null hypothesis. We therefore conclude that the knowledge extracted from the entropy-based model enhanced prediction.

Looking at the figures, we first notice that the staking model outperformed all other models. in a sense that, it had the best performing model for the majority of genes as shown in Fig. 1. Compared to the baseline model alone as shown in Fig. 2, it outperformed the baseline model in 292 genes, leaving only 14 genes model for the baseline. With the null hypothesis that there’s no difference in performance between the stacking model and the baseline model, the significant test showed a *p*-value of 4.4e−244, Therefore the null hypothesis can be rejected with a significance level of 0.01.

Moreover, we observed that each of the graph structure methods outperformed the baseline method, as they scored higher accuracy in more genes than the baseline. However, no graph structure method clearly outperformed the others. The best method was local linear embedding, which outperformed the base model in 194 cases in comparison to the base model’s 109 genes. Even common-neighbour based model, which is the model that has the weakest permanence, generally, outperformed baseline once they are compared as a pair. The pairwise comparison between the baseline and each of the methods clearly shows that all graph structure methods recorded higher accuracy compared to the baseline. This result is shown in Fig. 3.

### Analysis

Useful interpretable biological knowledge can be extracted by analysing the machine learning gene models. For example, the TP53 model revealed that the most important gene in enhancing predictions about it is ERBB3, which is a member of a tyrosine receptor kinase that is implicated in many cancers [13]. However, ERBB3 is not usually overexpressed in MCF-7 [14]. ERBB3 has a known role in therapy resistance as its downstream pathways include PI3K/AKT, MAPK/ERK and JAK/STAT [15–18]. Previous research has investigated the role of ERBB3 in tumour progression in mcf7, and concluded that there was an effect on the drugs targeting the er-$\alpha$ receptor once it coupled with a drug that targeted ERBB3 [19].

The ERBB3 model also plays a role in improving the predictions for CDK4, which is known to participate in inactivating RB gene in many cancers through loss of proliferation control [20]. CDK4 inhibitors such as Palbociclib and Ribociclib are known to prolong progression-free survival when combined with hormone therapy [21, 22].

Another example is in the CCNA2 model. CCNA2 is a cyclin and commonly overexpressed in many cancers [23]. Interestingly, the BIRC5 model is the model that affect CCNA2 gene predictions the most and vice versa. The BIRC5 gene is an apoptosis inhibitor, a member of the apoptosis pathway, and has no direct connection with CCNA2. However, it is known to be a good target for cancer therapy, as it is abundant in cancerous cells in the opposite of normal cells [24], yet there is no existing drug to target it available, or in clinical trials [25]. Both genes are associated with drug resistance [26, 27], and there is evidence that both genes are expressed in similar levels in mcf7 in response to different stimuli; however, they are rarely linked in literature [28].

Full details of gene associations based on the machine learning models is available in Additional file 1: Table S3.

## Discussion

Gene expression levels are subject to modification by many factors inside and outside the cell. This study has utilised mechanistic models that describe cellular signalling pathways to infer gene relationships. Although current signalling pathways are known to be incomplete, they provide a framework that describe the mechanisms controlling cell behaviour. Therefore, this knowledge is important in building models that predict this behaviour.

In this research we have exploited the fact that genes that share the same pathway or upstream regulator tend to have correlated behaviour. We have applied similarity heuristics, and embedding techniques, to generate input features for machine learning. Such heuristics reflect causal relationships in the cell that are likely to impact gene expression levels.

Graph-based methods are well suited for fast processing, they do not require parameters that are hard to obtain, in contrast of simulation-based and equation-based models. They are also arguably more general. However, graphs only capture the structure of signalling pathways, they do not capture the dynamics of the network, nor include pathway context. They therefore only represent a partial and simplistic picture of the actual phenomenon. Therefore, one way to improve the predictions would be to expand the model to include more aspects of the biological system, such as dynamics, or the contexts of the models. Methods that capture the causality of the system are likely to improve the performance of predicting gene expression levels even further.

## Methods and data

### Data

The data was taken from the Library of Integrated Network-based Cellular Signatures Phase 2 (LINCS), which measured the responses of different cell lines to different drug perturbations [29]. The data contains the expression levels for around 1000 genes as the ground truth, and associated experimental conditions: drug-related data, such as drug dosages, time point and properties of the drugs used to perturb the cells; cell line details, such as the source of cell lines, cancer types; and general patient data.

The data was first pre-processed by deleting missing values, fixing the inconsistency problem in some features, and deleting duplicated values. Then expression leves were normalised by scaling the data to be in the range from 0 to 1. We then we randomly chose 300 genes to predict from the MCF-7 cell line data. To keep track of how features were used in this study, we will call these the standard features to distinguish them from features generated by the mechanism models, which will be added later.

The mechanism-based models were taken from the Kyoto Encyclopedia of Genes and Genomes (KEGG) [30]. We focused on the declarative information in KEGG, and extracted it as undirected graphs. The knowledge included genes associations with other genes within pathways, pathways memberships and drugs associations.

Mutations in several cell lines were extracted from COSMIC [31], these were then added to the model as ‘super nodes’ to enhance the amount of knowledge that could be extracted from the graph. Among the 300 genes selected for this study, only 177 genes have known direct connection with other genes. Which means that 123 genes don’t have any direct neighbours, and therefore, they are not part of the original graph. However, there is another type of knowledge associated with these genes, such as them being part of a specific pathway or a target for a specific drug. Therefore, we used this knowledge to fill the gaps as we add pathways and drugs and cell lines as ‘super nodes’ that have an association with these genes.

### Methods

**Fig. 4:**
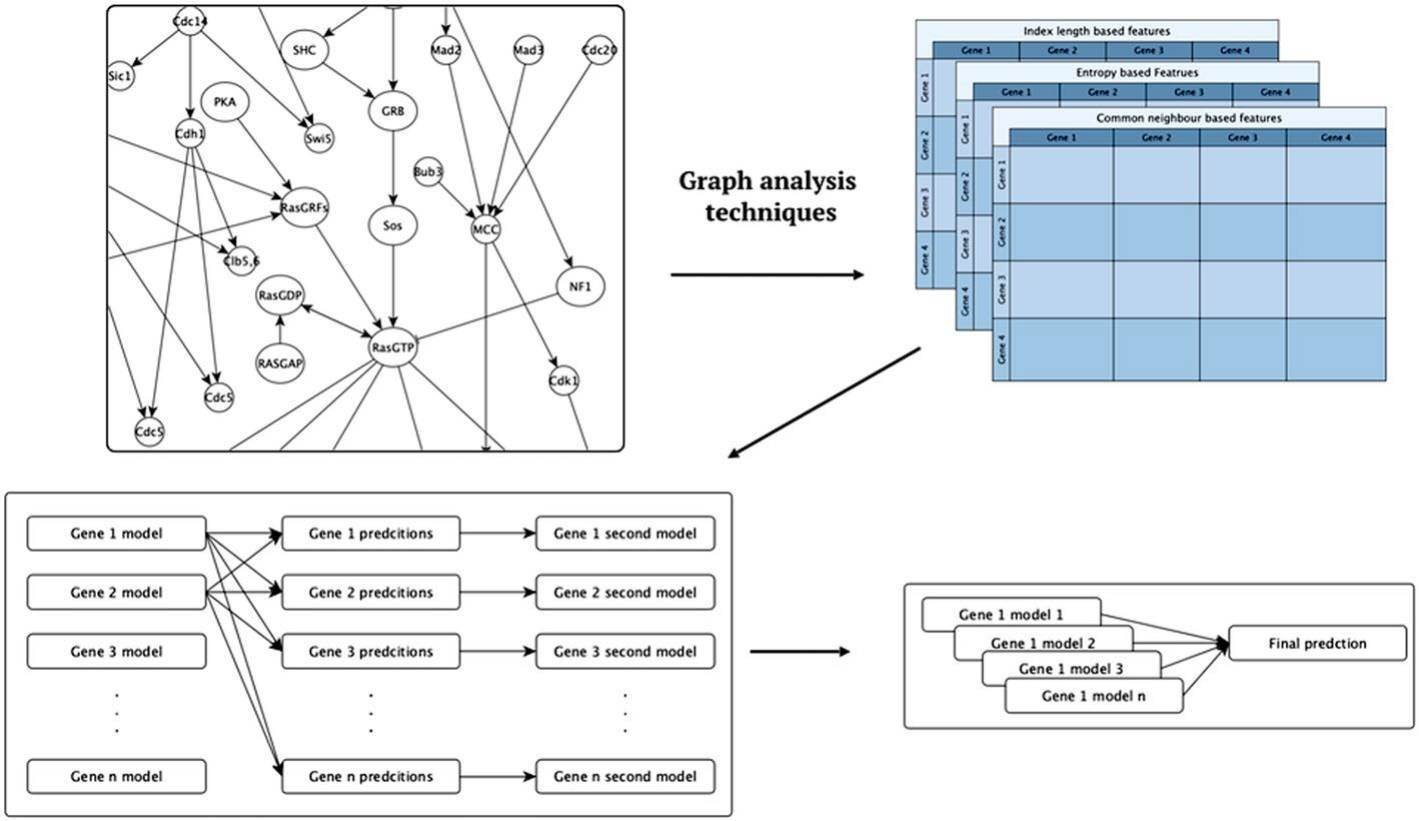
The general workflow describing the integration of signalling pathways models into the machine learning model

The signalling pathways were represented as graphs. Several graph processing methods were used in this study: three similarity heuristics, and four embedding methods. Graph processing was used to generate gene similarity features (attributes, descriptors), these were utilised by the machine-learning methods. Figure 4 shows the general workflow, explained in detail below.

The first step was to extract features from the mechanistic model, represented as a graph, where each gene was treated as a node connected to other genes belonging to the same pathway. The drugs were represented as nodes to show the influence they had on genes and pathways in general. In this approach, if a drug affects a specific gene, it might influence all genes linked to that gene. Few known targets were found for each drug in the databases, which is reasonable, as drugs are generally designed to be very specific. However, treating a cell with a single drug usually causes a cascade of changes due to the high connectivity of cellular networks. Therefore, our representation could capture expected changes in the behaviour of some genes when they are not the drug’s direct target. Pathways were represented as nodes that connected the genes belonging to them, to deal with the expected gaps in the models that arise from our incomplete bioinformatic knowledge.

We formalised the problem as follows: let *G* be the signalling pathways model, *G* = (*V*, *E*) , where *V* are the members of the signalling network, and *E* are edges connecting *V*. The goal is to learn vector *X* $\in ^{v\times s}$, where *s* is the number of latent dimensions. Then *X* is used in training the machine learning models. We implemented the machine learning method multi-target regressor stacking (MTRS) [32]. This learning algorithm consisted of two stages. In the first stage, an independent model $h_j$ : *X* $\rightarrow$ *R* is learned for every single gene. In the second round, meta models $h_j^*$ :$X*Y$ $\rightarrow$ *R* are learned for each gene. The meta models were learned using transformed training set *D* = ($x_i^{*1}$,$y_j^1$),...,($x^{*n}$,$y_j^n$) where *x* = [$x_1^j \ldots x_n^j$, $\hat{y}_i^1 \ldots \hat{y}_i^m$] are meta features consisting of the original features in addition to predictions obtained for each gene from the first stage models of other genes.

To extract the relations between different nodes, it is required to find similarities between different genes based on graph structure. There are many heuristic methods that can be used to compute such gene similarities, such as those that detect local similarities, for example, the common neighbour, the Jaccard index [33], the preferential attachment [34] and resource allocation [35]. There are also methods that can identify similarities based on the global structure of the network, such as, the Katz index [36] and random walk [37]. Other methods include path-based methods, such as the shortest path [38] and the local path index [39]. An alternative approach is to use a local probabilistic approach, such as the relative entropy-based method [40].

An alternative approach is to use graph embedding techniques to project genes into lower dimensions, where every gene is represented by a vector describing its context as a node within the graph. In principle this preserves much of the structure of the graph by keeping nodes that share the same cluster close to each other. The embedding can be done based on different approaches. One type is graph embedding based on matrix factorization techniques such as HOPE [41] and Laplacian eigenmaps [42]. There are also methods based on a random walk with deep learning, such as deepwalk [43] and nod2vec [44]. There are also methods based on deep learning without random walks such as SDNE [45], and GCN [46]

In this work, the representation for each node was calculated based on a selected subset of methods: common neighbour, local path index and entropy-based similarity. The embedding was calculated using Laplacian eigenmaps, locally linear embedding, deepwalk, and nod2vec.

#### Similarity measures

*The common neighbour method* In this the similarity score between two genes is defined by their shared neighbours in the signalling network. The heuristic is that if two genes share many neighbours, they are likely to be similar and affect the same function of the cell. The common neighbour score is computed by the following equation:

1 $$\begin{aligned} S = | \Gamma x \cap \Gamma y | \end{aligned}$$

In this equation, *x* and *y* are nodes (genes) and $\Gamma x$ and $\Gamma y$ are the neighbours of each node. This results in features for each gene which utilised later as features for the learning process.

*The local path index method* This similarity measure represents a balance between the local and global measures. In this setting the similarity between two genes is based on the lengths of the paths that connect them and are defined as follows:

2 $$\begin{aligned} {S_{x,y} = \sum _{i=2}^{l} B^l .|paths_{x,y}^l|} \end{aligned}$$

In this equation, *B* is a free parameter, and *l* is the length of paths. The value *paths* is the number of paths of length *l* that connect the two genes. If *l* is equal to two then this will give a result equal to the common neighbour method. If *l* is set to ${\infty }$, then it will be equal to the Katz index, which is a global and computationally expensive similarity measure. In the case of local path index, *l* is usually set to 3, where it calculates all paths of length 3 between two genes due to algorithm complexity. Therefore, it is more general than the common neighbour method and it can be computed within a reasonable time limit.

*The relative entropy-based method* This similarity measure is based on considering a network’s local structure as information. It uses the relative entropy between two genes to measure the difference between them. If the difference is small then the two genes are similar, and vice versa. This method works by first examining the local network for each node by measuring its degree distribution. Each node, *i*, will be defined by the set $L_i(N, D)$, where *N* is the number of genes in the local network, and *D* is the degree distribution for each *k* of *N*. Then, the probability distribution for each gene, *P*(*i*), is calculated and used to get the relative entropy. This probability distribution is calculated as following:

$$\begin{aligned} p(i,k)=\left\{ \begin{array}{ll} \frac{D(k)}{\sum _{k=1}^m D(k)}& \quad k \le Degree(i) + 1 \\ 0 &\quad k > Degree(i) + 1 \\ \end{array} \right. \end{aligned}$$

In this equation, *D*(*k*) is the degree of gene *k* in the local network, *Degree*(*i*) is the degree of gene *i* and *m* is the largest degree in all networks plus 1. In order to calculate the relative entropy, the probability set for each node is reordered in decreasing order. Then, the relative entropy is calculated for each couple of genes as follows:

3 $$\begin{aligned} D_{KL}(P(i)||P(j)) = \sum _{k-1}^{m'} P(i,k)ln \frac{P(i,k)}{P(j,k)} \end{aligned}$$

In this equation, $m'$ is $m' = min(D(i),D(j))+1$. In the end, the similarity score, $S_{i,j}$, for genes *i* and *j* is calculated as follows:

4 $$\begin{aligned} S_{i,j} = D_{KL}(P(i)||P(j)) + D_{KL}(P(j)||P(i)) \end{aligned}$$

#### Graph embedding techniques

*Laplacian eigenmaps* This method projects a graph structure into a low-dimension representation based on the Laplacian concept of a graph. This representation preserves the node’s neighbourhood information by constructing a weighted graph and adding edges between ‘close’ nodes, which require a definition of closeness based on one of two techniques. The first technique is the k-nearest neighbour method, where nodes *i* and *j* have edges if *i* is one of the k-nearest neighbours of *j*. The second technique is to set a threshold ‘$\epsilon$’ that adds an edge between two nodes *i* and *j* if the squared distance between them is less than $\epsilon$. Both techniques have their pros and cons. In our study, we used the k-nearest neighbour method as it does not usually produce disconnected graphs like the latter method [42]. The second step of the algorithm is to add the weights to the edges using either a heat-kernel method or setting all connected nodes to 1 and unconnected nodes to 0. Finally, compute the eigenvalues and eigenvectors by solving the eigenvector problem:

5 $$\begin{aligned} {Lf = \lambda Df } \end{aligned}$$

In this equation *D* is the diagonal weight matrix and $D_{i,j} = \Sigma W_{i,j}$, $L = D-W$. Data is projected into n eigenvectors, where n is selected by the user.

*Locally linear embedding* The locally linear embedding method [47] assumes that each node can be represented as a linear combination of its neighbours. It attempts to minimize the difference between the actual data points and their reconstructions. The algorithm consists of three main steps [48]. First, it finds the neighbours for each node. These neighbours can be determined by any local metric, but they are usually determined based on the Euclidean distance of k neighbours. Then, the algorithm computes the weights, $W_{i,j}$, that minimize the following cost function:

6 $$\begin{aligned} { \epsilon (W_i) = \sum _{i} | \overrightarrow{X}_i - \sum _{j} W_{ij} \overrightarrow{X}_j | ^2 } \end{aligned}$$

Finally, the algorithm computes the vector, $Y_{i}$, best reflected by the weights $W_{i,j}$, minimising the following equation to its lowest non-zero eigenvectors:

7 $$\begin{aligned} { \Phi (Y) = \sum _i | \overrightarrow{Y}_i - \sum _{j} W_{ij} Y_{j} | ^2} \end{aligned}$$

*Deepwalk* Deepwalk is an embedding method that learns the latent representation of a graph based on random walks. It preserves neighbourhood information by using many random walks to represent nodes. Then, it processes the resulting walk as a sentence that maintains the context of the node. DeepWalk also adapts one of the most beneficial concepts of natural language processing models, which is the skip-gram model [49]. This model is the core of word2vec [50], which maps text to vectors in order to make it easier for the computer to process text. The context of the node is used by a neural network to preserve the structure by minimizing the following function:

8 $$\begin{aligned} {minimize -log Pr (\ { v_{i-w}, \ldots ,v_{i+w} \} | \Phi (v_i)}) } \end{aligned}$$

The resulting representation will capture the neighbourhood similarities, since close nodes will have similar representations.

*Node2vec* The Node2vec method is similar to deepWalk in that it depends on a skip-gram model. However, node2vec uses a biased random walk to capture the context of a node by considering its role in the graph and its communities. This is done by changing the policy that is followed to generate the walk from random to biased, where the algorithm enables the user to prefer the depth-first or breadth-first walks.

### Machine learning

To run the machine learning, the seven sets of learning features, which comprise the gene similarity features based on the above methods, were added in turn to the standard features. Then, these were used to train seven different models. MTRS was implemented using Python [51] and the library Scikit-learn [52]. This learning algorithm involves independently building a random forest model for each gene of 300 genes were selected to conduct this study. Random forests are widely used in bioinformatics applications due to their high accuracy and plausible interpretability, such as in [53]; in this research, the random forest was used to predict DNA N6-methyladenine sites. Also, in [54] random forest classifier was used to identify neuropeptides. In our study, we randomly split the data into testing and training sets, each model was trained using 10,000 examples for each gene, and tested on around 3000 examples. Three hundred trees were used to construct the random forest for each model, with no restrictions on tree depth or leaf nodes.

Finally, for each gene, different methods models were stacked into a final model. The baseline model is a random forest model trained as MTRS without the extra features generated from graph processing. The stacking method used was an ensemble method where predictions of different previously trained models are combined to improve prediction results. By using stacking, we take advantage of different methods altogether. Different ML models behave differently on different genes, and combining them together in a stacking model enables the formation of a model that performs well on the majority of genes [55]. There are different implementation approaches to stacking. Our implementation directly combines the predictions without using an extra meta model.

## Supplementary Information

**Additional file 1: Supplementary table 1.** The accuracy of the first round model. **Supplementary table 2.** The accuracy of the seven models is illustrated as follows: cn is the common neighbour model, lpi is local path index model, ent is the relative-entropy model, loc is the locally linear embedding model, egin is the laplacian eigenmaps embedding model, n2v is the node2vec model, and depwlk is the deepwalk model. **Supplementary table 3.** The top 5 genes participating in improving the predictions for each gene, the last column demonstrate the importance of the first gene.

## Data Availability

The data, models are all freely available. The data is available at LINCS data. All models were built using Scikit-learn, NetworkX except for Node2vec and Deepwalk.

## Abbreviations

ML: : Machine learning
SMILES: : Simplified molecular-input line-entry system
KEGG: : The Kyoto Encyclopedia of Genes and Genomes
COSMIC: : The Catalogue Of Somatic Mutations In Cancer
MTRS: : Multi-target regressor stacking
