# Supplementary material for: Improved prediction of gene expression through integrating cell signalling models with machine learning
Source: BMC Bioinformatics. 2022 Aug 6;23:323. doi: 10.1186/s12859-022-04787-8 (PMC9356471; doi:10.1186/s12859-022-04787-8)
Supplement: Supplementary file 1 — Additional file 1: Supplementary table 1. The accuracy of the first round model. Supplementary table 2. The accuracy of the seven models is illustrated as follows: cn is the common neighbour model, lpi is local path index model, ent is the relative-entropy model, loc is the locally linear embedding model, egin is the laplacian eigenmaps embedding model, n2v is the node2vec model, and depwlk is the deepwalk model. Supplementary table 3. The top 5 genes participating in improving the predictions for each gene, the last column demonstrate the importance of the first gene. [file 12859_2022_4787_MOESM1_ESM.pdf]

Improved Prediction of Gene Expression through Integrating Cell Signalling Models with Machine Learning: supplemental document

Nada Altaweraqi[1,2*] and Ross D. King [3,4,5]

** Correspondence: Nadaaltaweraqi@postgrad.manchester.ac.uk. [1] Department of Computer Science, University of Manchester,Manchester, UK. Full list of author information is available at the end of the article*

supplementary table 1. The accuracy of the first round model.

Gene	R2	Gene	R2	Gene	R2	Gene	R2
DDR1	0.085	RRAGA	-0.021	SDHB	0.048	HES1	0.078
RHOA	0.018	SMC4	0.318	GTF2A2	0.076	PSMG1	0.199
RNPS1	0.044	NIPSNAP1	0.139	CCNB2	0.412	DDB2	0.373
RPS6	-0.038	MYBL2	0.276	LIG1	0.403	CCNA2	0.437
CSRP1	-0.044	ELAVL1	0.027	EBP	0.038	EML3	0.028
XBP1	0.543	KIAA0100	0.025	WRB	0.155	PRAF2	0.123
SKP1	0.007	TP53	0.331	SYNE2	-0.04	MRPL19	0.097
CAPN1	0.141	PSME2	0.242	OXCT1	0.34	MNAT1	-0.062
CTSD	0.288	LBR	0.062	NNT	0.132	RAB4A	-0.073
MAT2A	0.122	SCCPDH	0.125	GAA	0.133	BCL2	0.061
STMN1	0.341	SMARCD2	0.079	SLC37A4	0.03	RFC2	-0.026
CIRBP	0.17	NET1	0.367	HPRT1	0.104	SLC25A13	-0.009
PSME1	0.254	HDAC2	0.038	FAH	0.1	MRPL12	0.134
PGAM1	0.014	HSPB1	0.073	POP4	0.07	CEBPA	0.026
STAT1	-0.01	CDC25B	0.319	CDC20	0.262	SLC5A6	-0.011
GNAS	-0.023	ATMIN	0.044	NUP88	0.035	AURKA	0.359
HIF1A	-0.012	PSRC1	0.29	MSH6	0.088	CCNH	0.002
PAICS	0.08	UBE2A	0.094	PIN1	-0.002	CDC45	0.033
HADH	0.257	COASY	-0.078	ETFB	0.01	ENOSF1	0.074
SMARCC1	-0.025	BIRC5	0.532	CRYZ	0.168	CDK2	0.343
PIP4K2B	0.014	ARHGAP1	0.246	UBE2C	0.489	ELOVL6	0.492
PFKL	0.009	CPNE3	0.034	LYPLA1	-0.001	CREB1	-0.102
PGRMC1	0.048	NUP62	0.087	TIMELESS	0.502	PPIC	0.068
MBNL1	0.081	PCM1	0.02	PAN2	0.006	BRCA1	0.162
PCNA	0.242	NUP93	0.055	RB1	0.068	CCDC85B	0.101
ILK	0.051	PLK1	0.162	TBP	-0.011	CCP110	0.013
SYPL1	0.045	CDK4	0.446	HAT1	-0.081	CDC25A	0.03
NUDCD3	0.051	HSD17B10	0.134	S100A4	0.195	NCK1	-0.1
TOP2A	0.543	CSK	-0.063	B4GAT1	0.278	TMEM5	0.013
EBNA1BP2	0.135	TCEAL4	0.364	ABCB6	0.153	MELK	0.188

SCP2	0.114	USP1	0.204	RFC5	0.159	CCNF	0.239
TMEM109	0.231	MPC2	0.287	CDK1	0.46	TCFL5	0.014
TRAP1	0.026	DECR1	0.19	TLE1	0.052	ICAM3	0.067
SOX4	0.143	ERBB3	0.234	PAFAH1B3	0.114	ATF5	0.036
SCRN1	0.087	PRSS23	0.465	DCK	0.167	CCNE2	-0.011
NFKBIA	0.215	IKBKAP	-0.035	DYNLT3	-0.036	LSM6	0.2
G3BP1	-0.001	GALE	0.509	BAMBI	0.354	ITGAE	0.069
RPA1	0.117	PRKCD	0.007	SLC35A1	0.4	SGCB	-0.036
MCM3	0.378	S100A13	0.445	ITGB1BP1	-0.009	PLS1	0.019
SMC1A	-0.025	NRIP1	0.244	TIMM17B	0.025	PCMT1	0.192
PWP1	-0.04	EAPP	0.024	UGDH	0.094	INPP4B	0.07
ICMT	0.208	POLR2K	0.108	EZH2	0.141	CHEK1	0.047
RUVBL1	0.205	NRAS	0.029	MYCBP	0.212	DAG1	0.111
GHR	0.168	EED	0.073	ASAH1	0.034	ZDHHC6	0.02
SCYL3	-0.013	GATA3	0.353	SMARCA4	0.074	TIMM9	0.123
POLG2	-0.073	CETN3	0.002	ATP5S	0.142	GMNN	0.216
NOLC1	0.075	TGFB3	0.254	PARP2	0.01	CDCA4	0.256
POLE2	0.344	HIST1H2BK	0.22	AKR7A2	0.238	NENF	0.114
RAD51C	0.116	ANXA7	0.03	SPDEF	0.543	DNAJC15	-0.015
KIF14	0.079	PUF60	-0.009	CCNB1	0.188	HEBP1	0.193
SLC35A3	0.003	CFLAR	0.018	ERBB2	-0.015	CD320	0.031
AKT1	0.253	RPL39L	-0.03	VDAC1	-0.095	CHMP4A	0.086
MUC1	0.199	CHEK2	0.187	GAPDH	-0.086	HEATR1	-0.11
CASP2	-0.117	HMG20B	0.097	CNDP2	0.082	CISD1	0.238
PARP1	0.176	TPM1	0.162	ADI1	0.161	SUV39H1	0.178
FDFT1	0.095	BDH1	0.482	TMEM50A	0.198	VPS28	-0.053
CCND1	0.042	LSM5	0.112	HACD3	0.031	KIF20A	0.346
OXA1L	-0.092	TXNDC9	-0.092	YKT6	0.437	METRN	0.029
CDC42	-0.018	HN1L	0.178	BZW2	-0.026	LAGE3	0.285
ADH5	-0.053	ZMIZ1	-0.068	EVL	0.036	PXMP2	0.274
CYCS	-0.017	PDS5A	-0.013	MIF	0.156	AMDHD2	0.155
COPS7A	0.046	XPO7	-0.001	IARS2	0.285	FBXO11	-0.1
GLOD4	0.09	COG4	0.014	KLHDC2	0.205	MTERF3	0.217
DLD	-0.068	TMEM97	0.071	STUB1	0.136	PSMD10	-0.023
IFRD2	0.034	TFDP1	0.22	ENOPH1	-0.054	TSEN2	0.089
TSPAN6	0.012	KAT6B	0.005	MRPS2	-0.078	PRR7	0.105
CDKN1B	-0.009	ADO	-0.03	NUP85	0.17	GTPBP8	-0.077
PRPF4	-0.031	JADE2	0.179	FIS1	0.012	DENND2D	-0.043
CBR1	-0.016	PCCB	0.077	NUSAP1	0.25	ARPP19	-0.1
NR2F6	0.212	KIAA1033	0.062	MRPS16	0.068	ATG3	-0.042
TSPAN4	0.38	RRP1B	0.047	NPDC1	-0.004	WDR61	0.091
PSIP1	0.035	THAP11	-0.008	DERA	0.11	CANT1	-0.021
AURKB	0.284	C2CD5	0.26	UBR7	0.102	FAM63A	0.095
RPA3	0.252	POLR2I	-0.047	ISOC1	0.188	ATF1	0.028
HDGFRP3	0.061	RPIA	0.289	CCDC92	0.09	TSTA3	0.092
RRS1	0.036	PLCB3	0.161	SCAND1	0.202		

supplementary table 2. The accuracy of the seven models is illustrated as follows: cn is the common neighbour model, lpi is local path index model, ent is the relative-entropy model, loc is the locally linear embedding model ,egin is the laplacian eigenmaps embedding model, n2v is the node2vec model, and depwlk is the deepwalk model.

Gene	baseline	cn	ib	ent	locallylinear	lap_egn	n2v	deepwalk	stacking
DDR1	0.45	0.4532	0.4516	0.4519	0.4517	0.4527	0.4515	0.4518	**0.4538**
RHOA	0.4643	0.4636	0.4633	0.4637	0.4652	0.4636	0.4648	0.4622	**0.4662**
RNPS1	0.4	0.3988	0.4021	**0.4043**	0.4001	0.4007	0.4006	0.4015	0.4036
RPS6	**0.4167**	0.4145	0.4146	0.4146	0.4128	0.4139	**0.4167**	0.4162	**0.4167**
CSRP1	0.1946	0.1933	0.193	0.1923	0.1939	0.1935	0.1935	**0.1969**	0.1964
XBP1	0.7518	0.7516	0.7511	0.7518	0.7517	0.7515	0.7502	0.7512	**0.7526**
SKP1	0.4822	0.4826	0.4829	0.483	0.4838	0.4834	0.4812	0.4824	**0.4846**
CAPN1	0.4592	0.4585	0.4595	0.4593	0.4601	0.458	0.4582	0.4581	**0.4627**
CTSD	0.6112	0.6114	0.6114	0.6122	0.6104	0.6114	0.6114	0.6107	**0.613**
MAT2A	0.4682	0.4695	0.4717	0.4698	0.4718	0.471	0.4701	0.4718	**0.4725**
STMN1	0.676	0.6759	0.677	0.677	0.6773	0.6776	0.6762	0.6771	**0.678**
CIRBP	**0.5163**	0.5138	0.5141	0.5127	0.5119	0.5135	0.5132	0.5121	0.5155
PSME1	0.6093	0.6103	0.6111	0.6116	0.611	0.6107	0.6115	0.6111	**0.6124**
PGAM1	0.2458	0.2486	0.247	0.2484	0.248	0.2479	0.2488	0.2454	**0.2508**
STAT1	0.2715	0.2731	0.2729	**0.2743**	0.2673	0.2689	0.2713	0.2674	0.2737
GNAS	0.2093	0.2114	0.2093	0.2094	0.2093	0.2084	0.2111	0.21	**0.2122**
HIF1A	0.3849	0.3884	0.3889	0.3895	0.3876	0.3894	0.3869	0.3864	**0.3901**
PAICS	0.5619	0.5634	0.5626	0.5641	0.5621	0.5646	0.563	0.5632	**0.5651**
HADH	0.5623	0.562	0.5628	0.5633	0.5626	0.5631	0.5619	0.5637	**0.5639**
SMARCC1	0.4122	0.4144	0.4134	0.4149	0.4149	0.4169	0.4141	**0.417**	0.4169
PIP4K2B	0.3865	0.3904	0.3913	**0.3925**	0.3894	0.3922	0.3899	0.3882	0.3924
PFKL	0.2845	0.2866	0.2883	0.2868	0.287	0.2868	0.286	**0.2887**	0.2886
PGRMC1	0.5239	0.5243	0.5252	0.5244	0.5254	0.5243	0.5255	0.5242	**0.527**
MBNL1	0.4416	0.4401	**0.4441**	0.4425	0.4436	0.444	0.4391	0.4436	0.444
PCNA	0.4139	0.4114	0.4113	0.412	0.4121	0.413	0.4138	0.4112	**0.4145**
ILK	0.4552	0.4547	0.4528	0.4515	0.4539	0.453	0.4545	0.454	**0.4554**
SYPL1	0.4451	0.4451	0.4476	0.4477	0.4479	0.4455	0.4448	0.4482	**0.4484**
NUDCD3	**0.4956**	0.4928	0.4936	0.4912	0.4935	0.4949	0.4932	0.4926	0.4951
TOP2A	0.7958	0.7948	0.7956	0.7948	**0.7966**	0.7949	0.7946	0.795	0.7964
EBNA1BP2	0.5359	0.5367	0.5368	0.5363	0.5377	0.5364	0.5366	0.537	**0.5389**
SCP2	0.4834	0.4861	0.4881	0.4871	0.4883	0.4875	0.485	0.4877	**0.4884**
TMEM109	0.5836	0.5846	0.5828	0.5815	0.5837	0.5808	0.5835	0.5833	**0.5849**
TRAP1	0.4347	0.4346	0.4326	0.4349	**0.4366**	0.4342	0.4353	0.4359	**0.4366**
SOX4	0.5297	0.5311	0.5313	0.5318	**0.535**	0.5318	0.5327	0.5314	0.5339
SCRN1	0.4654	0.4625	0.4625	0.4626	0.4639	0.4628	0.4648	0.4647	**0.4656**
NFKBIA	0.4707	0.472	0.4689	0.4711	0.4702	0.468	**0.4729**	0.4701	**0.4729**
G3BP1	0.2792	0.2768	0.2787	0.2787	0.2739	**0.2814**	0.2769	0.2746	0.28
RPA1	0.5569	0.5566	0.555	0.5536	0.5565	0.5555	0.5564	0.5575	**0.5581**
MCM3	0.6614	0.6621	0.6619	0.6608	0.6622	0.6621	0.6627	0.6607	**0.6631**

SMC1A	0.1879	0.1896	0.1931	0.1926	0.1921	0.1915	0.1895	**0.1942**	0.1938
PWP1	0.3422	0.3393	0.3403	0.3429	**0.343**	0.3405	0.3389	0.3406	**0.343**
ICMT	0.5028	0.5027	0.5034	0.5023	0.5022	0.5015	0.5018	0.5031	**0.5042**
RUVBL1	0.5557	0.5571	0.5564	0.5574	0.555	0.5567	0.5551	0.5575	**0.5581**
RRAGA	0.4428	0.4445	0.4458	0.4461	0.4457	0.4469	0.445	0.4425	**0.4471**
SMC4	0.6742	0.6743	0.6758	0.6752	0.6747	0.6747	0.6742	0.6751	**0.6763**
NIPSNAP1	0.5525	0.5531	**0.556**	0.5541	0.5543	0.5523	0.5531	0.554	**0.556**
MYBL2	0.5776	0.5772	0.5784	0.5797	**0.581**	0.58	0.579	0.579	0.5808
ELAVL1	0.4433	0.4446	0.4426	0.4433	0.442	0.4431	0.4431	0.4407	**0.4452**
KIAA0100	0.4004	0.3996	0.3992	0.4009	**0.4034**	0.4	0.4011	0.401	0.4031
TP53	0.5886	0.59	0.5923	0.5911	**0.5927**	0.5922	0.5884	0.5924	**0.5927**
PSME2	0.5581	0.5575	0.5563	0.5562	0.5588	0.5583	**0.5604**	0.5575	0.5596
LBR	0.5088	0.5101	0.5081	0.509	0.5099	0.51	0.5102	0.5101	**0.5113**
SCCPDH	0.483	0.4821	0.4826	0.4841	0.4857	0.4835	0.4824	0.4848	**0.4859**
SMARCD2	0.4616	0.4631	0.4627	0.4627	**0.4652**	0.4633	0.4601	0.4614	0.4645
NET1	0.6266	0.6274	0.6264	0.6256	0.6262	0.6267	0.6273	0.6265	**0.6278**
HDAC2	0.5316	0.532	0.5327	0.533	0.5337	0.5335	0.5332	**0.5354**	0.5352
HSPB1	**0.4742**	0.4707	0.4694	0.4706	0.4726	0.471	0.4732	0.4725	0.4737
CDC25B	0.5267	0.5273	0.5265	0.5279	**0.5293**	0.5283	0.5265	0.5283	0.5291
ATMIN	0.483	0.483	0.4849	0.4824	0.4838	0.4817	0.4838	0.4817	**0.4854**
PSRC1	0.5967	0.5962	0.5966	0.5955	0.5982	0.5972	0.5968	0.5985	**0.5986**
UBE2A	0.4857	0.4837	0.4835	0.4842	0.4838	0.4822	0.484	0.4824	**0.4862**
COASY	0.4036	0.4032	0.4045	0.4052	0.4025	0.4042	0.4034	0.403	**0.4055**
BIRC5	0.7816	0.7814	0.7809	0.7826	0.7823	0.7825	0.7811	0.7821	**0.7829**
ARHGAP1	0.6153	0.6143	0.6141	0.6144	0.6143	0.6153	0.6142	0.6145	**0.6161**
CPNE3	0.4638	0.4657	0.4655	0.465	0.4661	0.4667	0.467	0.4646	**0.4684**
NUP62	0.536	0.5364	0.5373	0.5368	**0.5392**	0.5359	0.5345	0.5388	0.5387
PCM1	0.4049	0.4026	0.4068	0.4051	0.4067	0.4069	0.4042	0.404	**0.4074**
NUP93	**0.4355**	0.4336	0.4275	0.4284	0.4315	0.431	0.4346	0.4317	0.4347
PLK1	0.3966	0.3959	0.3959	0.3954	0.3962	0.3973	0.3969	0.397	**0.398**
CDK4	0.6708	0.67	0.67	0.6702	0.669	0.6698	0.6699	0.6695	**0.6711**
HSD17B10	0.5422	0.5426	0.5407	0.5419	0.5427	0.54	0.54	0.5409	**0.5434**
CSK	0.4011	0.4026	0.4022	0.4015	0.4019	0.4039	0.4017	0.4006	**0.4044**
TCEAL4	0.651	0.6514	0.6515	0.6506	0.6517	0.6514	0.6515	0.6518	**0.6525**
USP1	0.585	0.5839	0.5833	0.5844	0.5841	0.5843	0.5838	**0.586**	0.5858
MPC2	0.6136	0.6123	0.6156	0.6146	0.6151	0.6138	0.613	**0.6162**	0.6159
DECR1	0.5276	0.5292	0.5283	0.5287	0.5281	0.5278	0.5288	0.5267	**0.5303**
ERBB3	0.4996	0.5002	0.4997	0.5008	0.4995	0.5005	0.4997	0.5009	**0.502**
PRSS23	0.6763	0.6775	0.676	0.6768	**0.6794**	0.6782	0.6767	0.6787	0.6791
IKBKAP	0.3325	0.3346	0.332	0.3339	0.3352	0.3342	0.3336	0.3314	**0.3361**
GALE	0.7267	0.7261	0.7262	0.7258	0.7271	0.7267	0.7272	0.7259	**0.7279**
PRKCD	0.4411	0.4405	0.4401	0.4411	0.4393	0.4408	0.4404	0.4399	**0.4424**
S100A13	0.7024	0.7026	0.703	0.7043	0.7043	0.7036	0.7034	0.7044	**0.7046**
NRIP1	0.4643	0.462	0.4636	0.4653	0.4653	0.4646	0.4617	0.4643	**0.4664**
EAPP	0.2905	0.2906	0.2923	0.293	0.292	0.291	0.2927	0.2893	**0.2941**
POLR2K	0.5375	0.5371	0.536	0.5369	0.5364	0.5385	0.5356	0.5363	**0.5387**
NRAS	0.5165	0.5161	0.5188	0.5178	0.5191	0.5176	0.5177	0.5167	**0.5198**

SDHB	0.5161	0.5161	0.5192	0.5176	0.5175	0.5187	0.5157	0.5176	**0.5196**
GTF2A2	0.4871	0.487	0.4874	0.4872	0.4893	0.4891	0.4876	0.4868	**0.4905**
CCNB2	0.6618	0.6644	0.6629	0.6641	0.6632	0.6644	0.6632	0.6629	**0.6651**
LIG1	0.671	0.6715	0.672	0.6715	0.6721	0.6715	0.671	0.6708	**0.6729**
EBP	0.4182	0.4159	0.4161	0.4171	0.4161	0.4177	0.4165	0.4152	**0.4191**
WRB	0.5282	0.5294	0.53	0.5293	0.5307	0.5304	0.5288	**0.5322**	0.5312
SYNE2	0.3888	0.3883	0.3903	0.3901	0.3889	0.39	0.3891	0.3884	**0.3914**
OXCT1	0.6251	0.6266	0.6249	0.6246	0.6263	0.6256	0.6256	0.6258	**0.6268**
NNT	0.4925	0.4908	0.4909	0.4911	0.4928	0.4919	0.4921	0.4916	**0.4939**
GAA	0.5445	0.5456	0.545	0.5449	0.5441	0.546	0.5453	0.5447	**0.5471**
SLC37A4	0.4011	0.4023	0.4009	0.398	0.3999	0.4019	0.4023	**0.403**	**0.403**
HPRT1	0.4113	0.4112	0.4107	0.4131	0.4136	0.4127	0.4106	0.4121	**0.4139**
FAH	0.4888	0.4882	0.4877	0.4887	0.4891	0.4887	0.4896	0.4903	**0.4914**
POP4	0.5105	0.5122	0.5111	0.5096	0.512	0.511	0.5113	0.513	**0.5132**
CDC20	0.5349	0.5357	0.535	0.536	**0.5377**	0.5368	0.5363	0.5365	**0.5377**
NUP88	0.4804	0.479	0.4772	0.4769	0.4804	0.4791	0.4812	**0.4818**	0.4813
MSH6	0.4968	0.496	0.497	0.4976	0.4971	0.4982	0.4971	0.4958	**0.4988**
PIN1	0.3268	0.3296	0.3306	0.3283	0.3301	0.3305	0.3303	**0.332**	0.3318
ETFB	0.4607	0.4609	0.4634	0.4638	0.46	**0.4645**	0.4605	0.4636	0.4642
CRYZ	0.5211	0.5216	0.5237	**0.5246**	0.5226	0.523	0.5225	0.5231	0.5244
UBE2C	0.751	0.751	0.7524	0.7524	**0.7538**	0.7514	0.752	0.7512	0.7533
LYPLA1	0.2788	0.2769	0.2799	0.2776	**0.2818**	0.2806	0.2765	0.2791	0.2812
TIMELESS	**0.7269**	0.7257	0.7244	0.7242	0.7258	0.7247	0.7264	0.7233	0.7265
PAN2	0.2264	0.2277	0.2297	0.2256	0.2268	**0.2329**	0.2289	0.2283	0.231
RB1	0.4562	0.4552	0.4565	0.4575	0.4571	0.4567	0.4554	0.4571	**0.4586**
TBP	0.4453	0.4462	0.4446	0.4447	0.4469	0.4442	0.4464	0.4468	**0.4476**
HAT1	0.1991	0.2	0.202	0.2022	0.2002	0.1971	0.2022	0.1991	**0.2031**
S100A4	0.5006	0.5005	0.4998	0.5018	**0.503**	0.5022	0.5011	0.5016	0.5029
B4GAT1	0.61	0.6096	**0.6135**	0.6116	0.6095	0.6121	0.6098	0.6121	0.6124
ABCB6	0.4977	0.4975	0.4979	0.5002	0.5012	**0.5018**	0.4973	0.5012	0.5017
RFC5	0.4189	0.4213	0.422	0.4226	0.4229	0.4224	0.4202	0.4223	**0.4232**
CDK1	0.6971	0.6971	0.697	0.697	0.6971	0.6974	0.6963	0.6977	**0.6984**
TLE1	0.3466	0.3472	0.3487	0.3493	0.3509	0.3484	0.3492	0.3495	**0.3511**
PAFAH1B3	0.5619	0.5607	0.5606	0.5609	0.5606	0.5611	0.5609	0.5623	**0.5629**
DCK	0.4165	0.4176	0.4187	0.4189	0.4158	0.4184	0.4186	0.4174	**0.4197**
DYNLT3	0.3763	0.3784	0.377	0.3725	0.3763	0.3748	0.378	**0.3787**	**0.3787**
BAMBI	0.5989	0.6003	0.5989	0.5996	**0.6024**	0.5994	0.6007	0.601	0.6018
SLC35A1	0.673	0.6743	0.6753	0.6758	0.6755	0.6755	0.6746	0.6754	**0.6762**
ITGB1BP1	0.1526	0.1527	**0.1585**	0.1554	0.1534	0.1529	0.1557	0.1513	0.1565
TIMM17B	0.3699	0.371	0.3745	0.3745	0.3728	**0.3751**	0.3734	0.3708	0.3749
UGDH	0.2253	0.2263	0.2262	0.2245	0.2289	**0.2296**	0.2231	0.2264	0.229
EZH2	0.4902	0.4907	0.49	0.4895	0.4881	0.489	**0.492**	0.4913	0.4919
MYCBP	0.5828	0.5823	0.5831	0.5836	0.5837	0.5825	0.5819	0.5837	**0.5847**
HES1	0.4417	0.4432	0.4437	0.4411	**0.4455**	0.4416	0.4412	0.4443	0.4452
PSMG1	0.518	0.5205	0.5197	0.522	0.5214	0.5222	0.5188	0.5222	**0.5224**
DDB2	0.6351	0.6347	0.6324	0.6343	**0.6361**	0.6325	0.6351	0.6339	0.6359
CCNA2	0.7343	0.7343	0.7339	0.7332	**0.7354**	0.7332	0.7344	0.7348	0.7352

EML3	0.4451	0.4439	**0.4479**	0.4454	0.4462	0.4449	0.4438	0.4436	0.4475
PRAF2	0.5112	0.5123	0.5137	0.5136	0.5143	0.5128	0.5109	0.5124	**0.5147**
MRPL19	0.4781	0.4782	0.4777	0.4797	0.4789	0.4797	0.4786	0.4782	**0.4802**
MNAT1	0.3133	0.3149	0.3136	0.315	0.3153	0.3144	0.3151	**0.3168**	0.3165
RAB4A	0.3676	0.3711	0.369	0.3697	0.3682	0.3692	0.3719	0.3706	**0.3721**
BCL2	0.4264	0.4259	0.4286	0.4265	0.4266	0.4287	0.4263	**0.4298**	0.4292
RFC2	0.3452	0.3429	0.3434	0.3417	**0.3466**	0.3439	0.3448	0.3447	0.3463
SLC25A13	0.2891	0.2882	0.2884	**0.2908**	0.2885	0.2854	0.2877	0.2896	0.2906
MRPL12	0.5619	0.5623	0.5618	0.5602	0.563	0.5621	0.5636	0.5616	**0.5641**
CEBPA	0.4241	0.4251	0.4274	0.4287	0.429	0.4288	0.4269	0.4286	**0.4297**
SLC5A6	0.2991	0.2976	0.3005	0.2998	0.2971	0.2978	0.2993	0.3002	**0.3013**
AURKA	0.6638	0.6641	0.6651	**0.6663**	0.6653	0.6635	0.6644	0.6629	0.6659
CCNH	0.4035	0.4004	0.4032	0.4044	0.4041	0.4058	0.404	**0.4082**	0.4066
CDC45	0.2986	0.3022	0.3001	0.2987	0.3009	0.3	0.3016	0.2985	**0.3028**
ENOSF1	0.4783	0.4804	0.4807	0.4811	0.4821	0.4805	0.4801	**0.4831**	0.4823
CDK2	0.6066	0.6065	0.6067	0.6067	0.6052	**0.6082**	0.6067	0.6067	0.6081
ELOVL6	0.7138	0.714	0.7129	0.7128	0.7138	0.7112	0.7124	0.7132	**0.7147**
CREB1	0.1637	0.1672	0.1681	0.17	0.166	0.1699	0.1685	0.1676	**0.1701**
PPIC	0.414	0.4145	0.4123	0.4137	0.4131	0.4134	0.4127	0.4148	**0.4157**
BRCA1	0.4585	0.4601	0.4592	0.4598	0.4596	0.4608	0.4612	0.4584	**0.4615**
CCDC85B	0.3908	0.3912	0.3921	0.3931	0.3931	0.3937	0.3917	0.3938	**0.3947**
CCP110	0.3567	0.359	0.3604	**0.3616**	0.3584	0.3595	0.3587	0.3589	0.3613
CDC25A	0.4217	0.4217	0.4224	**0.4253**	0.4233	0.4229	0.4207	0.4221	0.425
NCK1	0.2086	0.2097	0.2069	0.206	0.2083	0.2055	0.2082	0.2073	**0.2106**
TMEM5	0.3733	0.3737	0.3726	0.3752	0.3738	0.3729	0.3746	0.3743	**0.376**
MELK	**0.2727**	0.2695	0.2689	0.2689	0.2722	0.2651	0.272	0.2705	0.272
CCNF	0.5626	0.5636	0.5615	0.5628	0.5643	0.5634	0.5645	0.5634	**0.5649**
TCFL5	0.3565	0.3545	0.3564	0.3558	0.3563	0.3562	0.357	**0.359**	0.3587
ICAM3	0.5135	0.5141	0.5145	0.5152	0.5134	0.5141	0.5147	0.5124	**0.5162**
ATF5	0.3238	0.3225	0.3216	0.3221	0.3224	0.3241	0.323	0.3241	**0.325**
CCNE2	0.2928	0.2921	0.289	0.2929	0.2927	0.2941	0.2949	0.291	**0.2951**
LSM6	0.5487	0.5495	0.5494	0.55	0.5491	0.5488	0.5487	**0.5511**	0.5508
ITGAE	0.347	0.3461	0.3455	0.3485	0.349	0.3495	0.3439	0.3444	**0.3497**
SGCB	0.239	0.2411	0.2433	0.2426	0.2402	0.2403	0.2412	**0.246**	0.2439
PLS1	0.335	0.3363	0.3343	0.3339	0.335	0.3324	0.3339	0.336	**0.3369**
PCMT1	0.5858	0.5852	0.5861	0.5871	0.5862	**0.588**	0.5852	0.5858	0.5876
INPP4B	0.4622	0.4615	0.4618	0.46	**0.4647**	0.4614	0.4621	0.4602	0.4643
CHEK1	0.3956	0.3983	0.3976	0.3972	0.3973	0.3988	0.3988	0.3945	**0.3996**
DAG1	0.4767	0.4777	0.4762	0.4756	0.4763	0.4768	0.4774	0.4761	**0.4786**
GHR	0.4814	0.4833	0.4806	0.481	0.4834	0.4821	0.4829	0.4795	**0.4842**
SCYL3	0.275	0.2756	0.2803	0.2792	0.2786	0.28	0.2755	0.2776	**0.2806**
POLG2	0.1879	0.1835	0.1868	0.1847	**0.191**	0.1868	0.1877	0.1879	0.1898
NOLC1	0.4819	0.4836	0.4845	**0.4869**	0.4837	0.4855	0.4838	0.4818	0.4867
POLE2	0.6244	0.626	0.6266	0.6266	0.6259	0.6261	0.6253	0.6265	**0.6274**
RAD51C	0.3238	0.3246	0.3263	0.3261	0.3233	**0.3277**	0.3261	0.3262	0.3273
KIF14	0.2685	0.2662	0.2674	0.2669	0.2653	0.2651	**0.2688**	0.2657	0.2687
SLC35A3	**0.4422**	0.4409	0.4397	0.4391	0.4408	0.4383	0.4402	0.4399	0.4419

AKT1	0.5895	0.5901	0.591	0.589	0.5899	0.5883	0.5903	0.5898	**0.5915**
MUC1	0.5587	0.5559	0.558	0.5583	0.5586	0.5584	0.5576	0.5582	**0.5602**
CASP2	0.2185	0.2206	0.2189	0.2216	0.2199	0.2207	0.2199	**0.226**	0.223
PARP1	**0.5533**	0.5515	0.5505	0.5524	0.5514	0.5495	0.5517	0.5516	0.5532
FDFT1	0.4287	0.4301	0.4282	0.4296	**0.4325**	0.427	0.4306	0.4278	0.4319
CCND1	0.3906	0.3914	0.3908	0.3928	0.3928	0.3923	0.394	0.3919	**0.3946**
OXA1L	0.1624	0.1622	0.1576	0.1586	0.163	0.1625	0.162	**0.1648**	0.1636
CDC42	0.3164	0.3202	0.3193	0.3186	0.3186	0.321	**0.3227**	0.3204	0.3222
ADH5	0.2575	0.2561	0.2576	0.26	0.256	**0.2607**	0.2555	0.2586	0.2605
CYCS	0.2857	0.286	0.2861	0.2862	0.2837	**0.288**	0.2847	0.2858	0.2879
COPS7A	0.3624	0.3647	0.3635	0.3635	0.3618	0.3641	0.3647	0.3631	**0.366**
GLOD4	0.5245	0.5264	0.5279	0.5279	0.5286	0.5289	0.5281	0.5277	**0.5292**
DLD	0.241	0.241	0.2402	0.2393	0.2384	0.2417	0.239	0.2414	**0.2426**
IFRD2	0.2813	0.2864	0.285	0.2842	0.2851	0.2846	0.2838	0.2841	**0.2867**
TSPAN6	0.3665	0.3705	0.3707	**0.3731**	0.3701	0.3707	0.37	0.3714	0.373
CDKN1B	**0.2094**	0.2047	0.2051	0.2026	0.202	0.2058	0.2044	0.2055	0.2072
PRPF4	0.2239	0.223	0.2223	0.2202	0.2212	0.222	**0.225**	0.223	0.2248
CBR1	0.4013	0.4015	0.4038	0.405	0.403	0.4033	0.4029	**0.4059**	0.4051
NR2F6	0.4343	0.432	0.4323	0.4315	0.4323	0.4335	0.4324	0.4328	**0.4345**
TSPAN4	0.5372	0.5391	0.5377	0.5397	0.5372	0.538	0.5401	0.5389	**0.5402**
PSIP1	0.237	0.2374	0.2364	0.2333	0.2372	0.2359	0.2378	0.2368	**0.2393**
AURKB	0.5254	0.5261	0.5261	0.5257	0.526	0.526	0.5266	0.5251	**0.5277**
RPA3	0.5603	0.5592	0.5614	0.5607	0.5601	0.5605	0.5601	0.5607	**0.562**
HDGFRP3	0.3709	0.3711	0.371	0.3701	0.3723	0.3717	0.3714	0.3687	**0.3733**
RRS1	0.2586	0.2616	0.2624	0.2587	0.2596	0.2588	0.2606	0.2596	**0.2626**
EED	0.4031	0.405	0.4047	0.4038	0.4039	0.405	0.4049	0.4043	**0.4063**
GATA3	0.6088	0.6087	0.6071	0.6079	0.6086	0.6086	0.6069	**0.6101**	0.6098
CETN3	0.4343	0.4348	0.4377	0.4389	0.4373	**0.4391**	0.4351	0.4366	0.4389
TGFB3	0.5191	0.5196	0.5181	0.519	0.518	0.519	0.5192	0.5196	**0.5206**
HIST1H2BK	0.4033	0.4024	0.4035	0.4026	0.4016	0.4035	0.4024	0.4002	**0.4047**
ANXA7	0.5195	0.5181	0.5193	0.5198	0.5177	0.5182	0.5202	0.5181	**0.5211**
PUF60	**0.261**	0.2588	0.2574	0.2573	0.2587	0.2558	0.2583	0.2586	**0.261**
CFLAR	0.3329	0.3354	0.3395	**0.3417**	0.3359	0.3382	0.3335	0.3349	0.3391
RPL39L	0.4126	0.415	0.4149	0.4139	0.4139	0.4152	0.4128	0.4137	**0.4162**
CHEK2	0.4908	0.4903	0.4917	0.4915	**0.4937**	0.4904	0.4905	0.4893	0.493
HMG20B	0.3731	0.373	0.3752	0.3761	0.3731	0.3764	0.3741	0.3738	**0.3767**
TPM1	0.5297	0.5299	0.5308	0.5307	0.5301	0.5294	0.5294	0.5307	**0.5316**
BDH1	0.7226	0.7229	0.7214	0.7219	0.7207	0.7208	0.7226	0.7221	**0.7233**
LSM5	0.3134	0.3122	0.3122	0.3116	0.3138	0.3125	0.3136	0.312	**0.3146**
TXNDC9	0.2033	0.2011	0.2026	0.2002	0.2005	0.201	0.2026	0.2007	**0.2041**
HN1L	0.5504	0.5505	0.5472	0.5479	0.5499	0.5477	0.5491	**0.5506**	**0.5506**
ZMIZ1	0.366	0.365	0.3686	0.3661	0.3655	0.3678	0.3675	**0.3689**	0.3688
PDS5A	0.4265	0.4274	0.4296	0.4308	**0.4324**	0.4276	0.4297	0.4285	0.431
XPO7	0.3139	0.3155	0.3139	0.3134	0.3165	0.3162	0.3142	0.3167	**0.3175**
COG4	0.4244	0.4251	0.4221	0.4229	0.4258	0.4225	0.4235	0.424	**0.4261**
TMEM97	0.4558	0.455	0.456	0.4537	**0.4609**	0.4533	0.4566	0.4584	0.4578
TFDP1	0.5556	0.5546	0.5547	0.5561	0.5567	0.5547	0.5562	**0.5581**	0.5576

KAT6B	0.3658	0.3681	0.3699	0.3669	**0.3701**	0.3676	0.3672	0.3635	0.3699
ADO	0.2842	0.2865	0.2875	0.2843	0.2823	0.2853	0.2851	0.2861	**0.2876**
JADE2	0.4849	0.4846	0.4833	0.4849	0.4843	0.4843	0.4855	0.4835	**0.4866**
PCCB	0.2735	0.2781	0.2745	0.2748	0.2738	0.2737	0.2773	**0.2783**	0.2776
KIAA1033	0.1856	0.1873	0.1903	0.1892	0.1857	0.1909	0.187	**0.1927**	0.1907
RRP1B	0.4777	0.4796	0.4814	0.4794	0.4806	0.4789	0.4785	0.4795	**0.4818**
THAP11	0.3153	0.3166	0.3177	0.3182	0.3197	0.3178	0.316	0.3186	**0.3198**
C2CD5	0.4357	0.4349	0.4368	0.4365	0.437	0.4355	0.4365	0.4374	**0.4382**
POLR2I	0.2334	0.2326	0.232	0.2334	0.2313	0.2338	0.2309	0.233	**0.2346**
RPIA	0.5189	0.5185	0.5213	**0.5224**	0.5194	0.5219	0.5184	0.5189	0.5219
PLCB3	0.4166	0.419	0.4201	0.4171	0.4185	0.4189	0.419	0.4185	**0.4204**
ASAH1	0.4465	0.4457	0.4453	0.4464	**0.4487**	0.4461	0.447	0.4455	0.4485
SMARCA4	0.5055	0.505	0.503	0.5021	0.5034	0.5032	**0.5071**	0.5017	0.506
ATP5S	**0.4624**	0.4602	0.4576	0.4576	0.4579	0.458	0.4588	0.4572	0.4609
PARP2	0.3808	0.3812	0.3824	0.3822	0.3837	0.3805	0.3819	0.3816	**0.3838**
AKR7A2	0.4009	0.3979	0.3997	0.3982	0.3989	0.3994	0.3985	0.4003	**0.4013**
SPDEF	0.7371	0.7376	0.7384	0.7386	**0.7392**	0.738	0.7381	0.7369	0.739
CCNB1	0.3837	**0.386**	0.384	0.3805	0.3843	0.3828	0.383	0.3825	0.3855
ERBB2	0.3681	0.3698	0.3679	0.3678	0.366	0.3679	0.3669	0.3687	**0.3705**
VDAC1	0.14	0.1411	0.1391	0.14	0.1419	**0.1452**	0.1408	0.1395	0.1437
GAPDH	0.2432	0.2428	0.2388	0.2422	0.2413	0.2428	0.2429	0.2396	**0.244**
CNDP2	0.3714	0.3729	0.3733	0.3742	0.3735	0.3734	0.3733	0.3717	**0.3755**
ADI1	0.3571	0.3573	0.3555	0.3543	0.3557	0.3538	0.3554	0.3547	**0.3582**
TMEM50A	0.532	0.5311	0.5318	0.5294	0.5331	0.5297	0.5319	0.5293	**0.5332**
HACD3	0.2863	0.2859	0.2865	0.2875	0.2876	0.2887	0.2877	0.2863	**0.2897**
YKT6	0.733	0.7339	0.7338	0.7321	0.734	0.7347	0.7333	0.7337	**0.7351**
BZW2	0.3537	0.3557	0.3565	0.356	0.3545	0.3575	0.3532	0.3566	**0.3577**
EVL	0.4192	0.4176	0.4194	0.4186	0.418	0.4179	0.4194	0.4196	**0.4206**
MIF	0.4915	0.4931	0.4957	0.4951	0.4928	**0.4965**	0.492	0.495	0.4956
IARS2	0.4712	0.4698	0.4701	0.4704	**0.4728**	0.4701	0.4704	0.4691	0.4721
KLHDC2	0.5697	0.5711	0.5693	0.5686	0.5677	0.5677	**0.5717**	0.5694	0.5713
STUB1	0.3342	0.3356	0.3338	0.3358	0.3376	0.3374	0.3347	0.3351	**0.3377**
ENOPH1	0.2858	0.2885	**0.2917**	0.2897	0.2867	0.2885	0.2875	0.2911	0.2906
MRPS2	0.2632	0.2639	0.2603	0.2603	0.258	0.2616	0.2634	0.2629	**0.2642**
NUP85	0.424	0.4248	0.4216	0.4252	0.4233	**0.4274**	0.4219	0.4246	0.4258
FIS1	0.2984	0.2943	0.2992	0.2989	0.2984	0.2992	**0.3009**	0.299	0.3008
NUSAP1	0.4675	0.4699	0.4706	**0.4733**	0.4698	0.471	0.4693	0.469	0.4723
MRPS16	0.3476	0.347	0.3484	0.3494	0.3435	**0.3501**	0.3469	0.3443	0.3495
NPDC1	0.2553	0.258	0.2593	0.2561	0.2577	0.256	0.2571	**0.2608**	0.2603
DERA	0.3533	0.3531	0.3556	0.3563	0.3533	0.3516	**0.357**	0.356	**0.357**
UBR7	0.4379	0.4389	0.438	0.4394	0.4374	0.4371	0.4375	0.4363	**0.44**
ISOC1	0.3885	0.3892	0.3882	0.39	0.3887	0.3867	0.3895	0.3898	**0.3908**
CCDC92	0.3958	0.3974	0.3968	0.3966	0.3972	0.3971	0.3966	0.396	**0.3988**
SCAND1	0.4478	0.4465	0.4466	0.4467	0.4461	0.4467	0.448	0.4467	**0.4489**
ZDHHC6	0.3468	0.3465	0.3439	0.3469	0.3475	0.347	0.3467	0.3441	**0.3484**
TIMM9	**0.3882**	0.3871	0.3832	0.3826	0.3856	0.3834	0.3869	0.382	0.3875
GMNN	0.547	0.5464	**0.5503**	0.5491	0.549	0.5488	0.5476	0.5474	0.5498

CDCA4	0.5363	0.5355	0.5365	0.5371	0.5375	0.5353	0.5358	0.534	**0.538**
NENF	0.4988	0.4968	0.4976	0.4989	0.4942	0.4986	0.4975	0.4956	**0.4995**
DNAJC15	0.3155	0.3166	0.317	0.3154	**0.322**	0.3152	0.3158	0.3155	0.3196
HEBP1	0.4344	0.4352	0.4367	0.4352	0.4371	0.4357	0.4368	0.4367	**0.4376**
CD320	0.3378	0.3387	0.3382	0.3371	0.3388	0.3377	0.3384	0.3396	**0.3402**
CHMP4A	0.3617	0.3616	0.3607	0.3628	0.3639	0.3616	**0.365**	0.3642	0.3649
HEATR1	0.2344	0.2352	0.2364	0.2343	0.2365	0.2355	0.2367	0.2337	**0.2383**
CISD1	0.5626	0.561	0.5621	0.563	0.5633	0.5613	0.5611	0.5625	**0.5637**
SUV39H1	0.4118	0.4106	0.4075	0.4102	0.4122	0.4097	0.4105	0.4097	**0.4127**
VPS28	0.337	0.3366	0.3326	0.3348	**0.3386**	0.3352	0.3362	0.3385	0.3379
KIF20A	0.5461	0.5452	0.5433	0.544	0.5458	0.5455	0.5441	0.5448	**0.5467**
METRN	0.2418	**0.2464**	0.2441	0.2433	0.2415	0.2451	0.2421	0.2389	0.2461
LAGE3	0.5423	0.5429	0.5419	0.5408	0.5416	0.5424	0.5431	0.5409	**0.5439**
PXMP2	0.5113	0.5101	0.5115	0.5107	0.5098	**0.5127**	0.5116	0.5107	0.5126
AMDHD2	0.4398	0.4391	0.4399	0.4387	0.439	0.4383	0.4398	**0.4418**	0.4414
FBXO11	0.1828	0.1869	0.1878	0.1869	0.1877	0.1866	0.1882	**0.1937**	0.1895
MTERF3	0.4058	0.4035	0.4056	0.4037	0.4048	0.4042	0.4032	0.4029	**0.406**
PSMD10	0.3725	0.3724	0.3742	0.3708	0.3703	0.3707	0.3733	0.3739	**0.3745**
TSEN2	0.4133	0.4154	**0.4179**	0.4155	0.4165	0.4146	0.4152	0.4151	0.4173
PRR7	0.4656	0.4683	0.4693	**0.4715**	0.4686	0.4688	0.4658	0.4695	0.4703
GTPBP8	0.2136	0.2129	0.2159	0.2158	0.2135	**0.2171**	0.2134	0.2161	**0.2171**
DENND2D	**0.3045**	0.3033	0.3013	0.3023	0.3004	0.3019	0.3025	0.3008	**0.3045**
ARPP19	0.2112	0.2096	0.2116	0.2105	0.2101	0.2066	0.2083	0.2126	**0.2128**
ATG3	0.2906	0.2902	0.2919	0.2908	0.2909	0.2893	0.289	0.2883	**0.293**
WDR61	0.4329	0.432	0.4339	0.4348	0.4332	0.4323	0.4319	**0.4373**	0.4353
CANT1	0.2319	**0.2347**	0.231	0.2312	0.232	0.2324	0.2312	0.2325	0.2346
FAM63A	0.4159	0.4157	0.4125	0.4156	0.4148	0.4178	0.417	**0.4208**	0.4183
ATF1	0.4881	0.4888	0.4901	0.4905	0.4884	0.4902	0.4886	0.4901	**0.4911**
TSTA3	0.4897	0.4897	0.4908	0.491	0.4905	**0.4925**	0.4891	0.4917	0.4924

supplementary table 3. The top 5 genes participating in improving the predictions for each gene,the last column demonstrate the importance of the first gene.ß

index	gene 1	gene 2	gene 3	gene 4	gene 5	gene importance
**DDR1**	SCRN1	NNT	ERBB3	NIPSNAP1	BCL2	0.211
**RHOA**	SKP1	ELAVL1	SCP2	PAICS	NIPSNAP1	0.29
**RNPS1**	RRP1B	GLOD4	TMEM109	NUP62	SYPL1	0.194
**RPS6**	S100A13	SLC35A1	CPNE3	PSME1	NRAS	0.133
**CSRP1**	GALE	HSD17B10	GAPDH	STUB1	CHMP4A	0.072
**XBP1**	SPDEF	NET1	NRAS	HN1L	PRSS23	0.297
**SKP1**	NRAS	RHOA	LAGE3	ANXA7	TMEM50A	0.247
**CAPN1**	MUC1	THAP11	HIST1H2BK	S100A4	HES1	0.544
**CTSD**	AKT1	PRSS23	NPDC1	EML3	PRR7	0.279
**MAT2A**	CDC25A	AURKB	MSH6	TSEN2	NR2F6	0.234
**STMN1**	BIRC5	TIMELESS	EZH2	TOP2A	ANXA7	0.259
**CIRBP**	GAA	ARHGAP1	DDB2	TCEAL4	PRAF2	0.249
**PSME1**	DECR1	NIPSNAP1	PSME2	SKP1	NRAS	0.335
**PGAM1**	PAICS	HSD17B10	MYCBP	TFDP1	MIF	0.158
**STAT1**	UBE2A	NNT	ARHGAP1	SYNE2	RPA1	0.151
**GNAS**	GAA	SMARCA4	RPA1	NIPSNAP1	ENOSF1	0.082
**HIF1A**	CCNH	TCEAL4	RB1	TIMM17B	CTSD	0.196
**PAICS**	HDAC2	PSMG1	ICAM3	LAGE3	BDH1	0.336
**HADH**	SCP2	CISD1	CHEK2	OXCT1	S100A13	0.124
**SMARCC1**	MBNL1	SYNE2	HN1L	NOLC1	CNDP2	0.18
**PIP4K2B**	DAG1	NNT	B4GAT1	NIPSNAP1	GAA	0.201
**PFKL**	GAA	LIG1	ELAVL1	PARP1	MIF	0.124
**PGRMC1**	RPA1	SDHB	HN1L	UBE2A	ANXA7	0.308
**MBNL1**	SMC4	SMARCC1	LBR	GLOD4	HN1L	0.223
**PCNA**	CCNA2	PPIC	UBE2C	HPRT1	UGDH	0.288
**ILK**	TSTA3	NUDCD3	PSME2	S100A4	TPM1	0.101
**SYPL1**	PIP4K2B	NNT	PDS5A	RPS6	HDAC2	0.22
**NUDCD3**	ENOSF1	TSTA3	PIP4K2B	RAB4A	SKP1	0.165
**TOP2A**	UBE2C	CDK1	YKT6	KIF20A	SMC4	0.352
**EBNA1BP2**	SDHB	NOLC1	NUP93	RPIA	TPM1	0.293
**SCP2**	NNT	RHOA	CRYZ	NRAS	DECR1	0.24
**TMEM109**	TIMELESS	PAFAH1B3	MCM3	CDK4	RFC2	0.362
**TRAP1**	MRPL12	TSTA3	SMARCA4	RUVBL1	PAICS	0.114
**SOX4**	TLE1	NFKBIA	GATA3	NET1	BAMBI	0.147
**SCRN1**	DDR1	PSRC1	TPM1	RB1	CHEK1	0.245
**NFKBIA**	SOX4	DAG1	GAA	GATA3	HSD17B10	0.288
**G3BP1**	TMEM97	POLR2K	CPNE3	CDCA4	USP1	0.116
**RPA1**	PGRMC1	TIMELESS	SMARCA4	ATMIN	SYNE2	0.204
**MCM3**	TIMELESS	TMEM109	SMC4	CDK2	NUP85	0.578
**SMC1A**	XPO7	TIMELESS	OXCT1	MAT2A	GAPDH	0.067
**PWP1**	MRPL19	EZH2	TBP	NOLC1	EBNA1BP2	0.112
**ICMT**	TIMELESS	TMEM109	RPA1	PARP1	AKT1	0.171

**RUVBL1**	MYBL2	TFDP1	PAFAH1B3	SDHB	TSEN2	0.32
**RRAGA**	PRAF2	SCRN1	CCDC92	GALE	TPM1	0.094
**SMC4**	AURKA	CCNA2	YKT6	TOP2A	NUP62	0.413
**NIPSNAP1**	PSME1	BDH1	PIP4K2B	CDK4	RPA1	0.249
**MYBL2**	TMEM109	RUVBL1	TFDP1	BIRC5	TIMELESS	0.137
**ELAVL1**	PAICS	RHOA	HACD3	SCRN1	RB1	0.355
**KIAA0100**	DAG1	HSD17B10	ENOSF1	HN1L	GAA	0.084
**TP53**	ERBB3	CDK4	DDB2	NR2F6	JADE2	0.309
**PSME2**	PSME1	HSD17B10	SDHB	MYCBP	ILK	0.18
**LBR**	SMC4	AURKA	EZH2	HDAC2	PCMT1	0.106
**SCCPDH**	INPP4B	GAA	SDHB	ANXA7	DECR1	0.265
**SMARCD2**	TSTA3	COPS7A	COG4	SMARCA4	AKT1	0.184
**NET1**	XBP1	TP53	GATA3	BAMBI	GAA	0.357
**HDAC2**	PAICS	NRAS	POP4	GALE	PSMD10	0.277
**HSPB1**	S100A13	SMARCD2	MIF	RPS6	ILK	0.067
**CDC25B**	OXCT1	YKT6	TSPAN4	SLC35A1	MSH6	0.222
**ATMIN**	DAG1	NUP62	PCM1	RRP1B	PCMT1	0.051
**PSRC1**	BIRC5	AURKA	AURKB	SCRN1	YKT6	0.299
**UBE2A**	CPNE3	ANXA7	EBNA1BP2	PGRMC1	EAPP	0.26
**COASY**	MYCBP	TSTA3	SKP1	GAPDH	ICAM3	0.079
**BIRC5**	CCNA2	POP4	YKT6	NUSAP1	AURKA	0.51
**ARHGAP1**	AKT1	PLCB3	LIG1	TIMELESS	TMEM109	0.183
**CPNE3**	UBE2A	SKP1	PCMT1	ATF1	RPS6	0.252
**NUP62**	SMC4	JADE2	PAICS	HN1L	LIG1	0.345
**PCM1**	PDS5A	HN1L	NUP62	XPO7	ENOSF1	0.108
**NUP93**	EBNA1BP2	FAH	CCNH	GATA3	NOLC1	0.32
**PLK1**	BRCA1	AURKA	BIRC5	CDC20	KIF14	0.163
**CDK4**	ERBB3	BDH1	TMEM109	TP53	PSME1	0.274
**HSD17B10**	SDHB	SMARCA4	DAG1	NIPSNAP1	GALE	0.351
**CSK**	AKT1	CEBPA	COG4	MRPL12	PRKCD	0.168
**TCEAL4**	DECR1	NR2F6	ERBB3	CDK4	PSME1	0.184
**USP1**	CDCA4	SMC4	YKT6	ATF1	NUP62	0.313
**MPC2**	GTF2A2	MYCBP	ELOVL6	RPA3	ITGAE	0.313
**DECR1**	PSME1	TCEAL4	DDB2	TMEM50A	DYNLT3	0.279
**ERBB3**	NNT	PIP4K2B	GATA3	SOX4	KLHDC2	0.152
**PRSS23**	SPDEF	CTSD	XBP1	NRIP1	EZH2	0.33
**IKBKAP**	TIMELESS	RPA1	EZH2	AKT1	TSEN2	0.18
**GALE**	DDB2	CDC25B	ABCB6	TSPAN4	MRPL12	0.138
**PRKCD**	PIP4K2B	CSK	GAA	BAMBI	SYPL1	0.236
**S100A13**	ICMT	PSME1	TSPAN4	SLC35A1	DECR1	0.187
**NRIP1**	XBP1	C2CD5	SOX4	GATA3	PRSS23	0.266
**EAPP**	SLC35A1	ATF1	UBE2A	TSPAN6	PSME1	0.157
**POLR2K**	NRAS	SKP1	MYCBP	CRYZ	CCNH	0.318
**NRAS**	HDAC2	POLR2K	SKP1	CRYZ	LYPLA1	0.309
**SDHB**	ANXA7	HSD17B10	PGRMC1	PAICS	EBNA1BP2	0.316
**GTF2A2**	POP4	MPC2	CRYZ	SLC35A1	DNAJC15	0.357
**CCNB2**	CCNA2	BIRC5	TOP2A	AURKA	KIF20A	0.441

**LIG1**	ARHGAP1	TIMELESS	S100A13	GAA	RPA1	0.273
**EBP**	PAICS	FDFT1	LAGE3	TMEM97	ICAM3	0.262
**WRB**	PAICS	CISD1	PCMT1	MYCBP	NIPSNAP1	0.185
**SYNE2**	RPA1	SMARCC1	TIMELESS	MSH6	PDS5A	0.077
**OXCT1**	PARP1	TIMELESS	NENF	GHR	ELOVL6	0.278
**NNT**	SCP2	RPA1	TOP2A	SYPL1	PIP4K2B	0.277
**GAA**	PRAF2	KIAA0100	NPDC1	LIG1	PIP4K2B	0.307
**SLC37A4**	NUDCD3	CDK4	ARHGAP1	PRR7	WRB	0.147
**HPRT1**	DCK	NRAS	MYCBP	WRB	PAICS	0.123
**FAH**	PAICS	NUP93	LAGE3	HSD17B10	GAA	0.285
**POP4**	GTF2A2	HDAC2	NUP88	ANXA7	BIRC5	0.354
**CDC20**	AURKA	BIRC5	PLK1	YKT6	EBNA1BP2	0.357
**NUP88**	YKT6	EZH2	MRPL12	POP4	PARP2	0.166
**MSH6**	CDC25A	SMC4	TIMELESS	NUP62	PDS5A	0.176
**PIN1**	HSD17B10	GALE	PAFAH1B3	GHR	HMG20B	0.186
**ETFB**	PAFAH1B3	TSTA3	COG4	PIP4K2B	BDH1	0.288
**CRYZ**	NRAS	SLC35A1	ANXA7	POLR2K	DNAJC15	0.305
**UBE2C**	TOP2A	YKT6	CDK1	AURKA	CCNA2	0.467
**LYPLA1**	NRAS	SKP1	POLR2K	RPA3	ICAM3	0.154
**TIMELESS**	MCM3	RPA1	LIG1	BIRC5	TMEM109	0.423
**PAN2**	TP53	ERBB3	CDKN1B	PRPF4	POLG2	0.075
**RB1**	TGFB3	SCRN1	CISD1	CCNH	HADH	0.154
**TBP**	HDAC2	AURKA	ANXA7	ATF1	EAPP	0.098
**HAT1**	RPA3	POLR2K	DNAJC15	CYCS	TFDP1	0.11
**S100A4**	SLC35A3	ILK	MPC2	CCDC92	KLHDC2	0.102
**B4GAT1**	BDH1	ICAM3	ICMT	PRSS23	SPDEF	0.346
**ABCB6**	GALE	HSD17B10	ANXA7	PARP1	GAA	0.29
**RFC5**	BRCA1	BIRC5	POLE2	CDK1	PSMG1	0.154
**CDK1**	TOP2A	UBE2C	BIRC5	NRAS	CCNA2	0.508
**TLE1**	SOX4	ATMIN	ERBB2	GAA	GATA3	0.178
**PAFAH1B3**	ETFB	TMEM109	BDH1	BIRC5	LAGE3	0.292
**DCK**	HPRT1	STMN1	POLE2	CDK1	PSMG1	0.135
**DYNLT3**	DECR1	PSME1	POLR2K	CPNE3	B4GAT1	0.107
**BAMBI**	NET1	SOX4	KLHDC2	PRKCD	SLC35A1	0.304
**SLC35A1**	KLHDC2	S100A13	ELOVL6	ATF1	EAPP	0.17
**ITGB1BP1**	SDHB	ANXA7	TSTA3	SMARCD2	PCNA	0.073
**TIMM17B**	PSME2	RPL39L	ATG3	PRR7	HIF1A	0.169
**UGDH**	PCNA	SCCPDH	NET1	INPP4B	CCND1	0.042
**EZH2**	STMN1	CDC25A	NUP88	ZDHHC6	SMC4	0.203
**MYCBP**	SDHB	POLR2K	LAGE3	SKP1	MRPL12	0.181
**HES1**	KLHDC2	CCDC92	ZMIZ1	STMN1	ASAH1	0.141
**PSMG1**	PAICS	ANXA7	HDAC2	USP1	CDCA4	0.356
**DDB2**	GAA	DECR1	S100A13	CIRBP	DENND2D	0.284
**CCNA2**	BIRC5	SMC4	PAICS	AURKA	TOP2A	0.499
**EML3**	TMEM109	AKT1	CSK	FIS1	CTSD	0.197
**PRAF2**	GAA	NIPSNAP1	SKP1	ASAH1	PSME1	0.346
**MRPL19**	MRPL12	EBNA1BP2	SDHB	TMEM109	TIMELESS	0.123

**MNAT1**	POLR2K	RPA3	PCMT1	ATF1	NRAS	0.093
**RAB4A**	MYCBP	TMEM5	PSME1	NUDCD3	GAPDH	0.064
**BCL2**	SCRN1	DDR1	EVL	OXCT1	ICMT	0.141
**RFC2**	PAFAH1B3	TMEM109	MYBL2	NUP93	BIRC5	0.224
**SLC25A13**	SMARCA4	SMC4	OXCT1	MBNL1	PARP1	0.099
**MRPL12**	PAICS	GALE	SMC4	MYCBP	CCND1	0.342
**CEBPA**	MRPL12	CSK	GALE	DAG1	NUP62	0.102
**SLC5A6**	SMARCA4	GAPDH	CSK	MELK	SMARCD2	0.169
**AURKA**	SMC4	CCNA2	YKT6	CDC20	TOP2A	0.383
**CCNH**	CISD1	POLR2K	RB1	NUP93	ATG3	0.152
**CDC45**	CDK2	DERA	CDC25A	RUVBL1	POLE2	0.136
**ENOSF1**	SMARCA4	SDHB	PIP4K2B	GAA	PAICS	0.277
**CDK2**	MYBL2	CDK1	NUSAP1	ICMT	MCM3	0.142
**ELOVL6**	SPDEF	FDFT1	CCNB2	MRPL19	HADH	0.428
**CREB1**	HDAC2	MTERF3	ITGAE	HES1	SCRN1	0.048
**PPIC**	MYCBP	PRAF2	PSMD10	RHOA	LIG1	0.159
**BRCA1**	SUV39H1	TIMELESS	MCM3	CDC25A	MYBL2	0.103
**CCDC85B**	TSTA3	TRAP1	CCND1	PRR7	ENOSF1	0.176
**CCP110**	MBNL1	SYNE2	TIMELESS	TMEM109	USP1	0.122
**CDC25A**	TIMELESS	BRCA1	TMEM109	SUV39H1	EED	0.256
**NCK1**	TBP	NRAS	ATF1	PSMD10	CDKN1B	0.121
**TMEM5**	PRR7	EBNA1BP2	RPS6	TSTA3	ENOSF1	0.079
**MELK**	USP1	SLC5A6	BIRC5	SMC4	CCNA2	0.088
**CCNF**	YKT6	CDCA4	B4GAT1	MSH6	PCMT1	0.456
**TCFL5**	GAA	PIP4K2B	TSPAN4	SKP1	CISD1	0.119
**ICAM3**	BDH1	PAICS	LAGE3	B4GAT1	MRPS16	0.186
**ATF5**	AURKA	PSMG1	MYBL2	MAT2A	TIMELESS	0.075
**CCNE2**	CDK2	CDC25A	CCNH	BRCA1	CDC45	0.121
**LSM6**	GMNN	S100A13	ATF1	RPA3	POLE2	0.198
**ITGAE**	GTF2A2	GMNN	CRYZ	ADI1	S100A4	0.176
**SGCB**	SKP1	POP4	RHOA	BIRC5	PFKL	0.101
**PLS1**	NNT	VPS28	CCDC92	TPM1	SLC35A3	0.143
**PCMT1**	GHR	POLR2K	LBR	CRYZ	SPDEF	0.093
**INPP4B**	SCCPDH	ERBB2	LBR	DENND2D	NET1	0.289
**CHEK1**	AURKB	OXCT1	BRCA1	HADH	SCRN1	0.137
**DAG1**	PIP4K2B	HN1L	KIAA0100	HSD17B10	SKP1	0.208
**GHR**	OXCT1	PCMT1	MPC2	DAG1	CEBPA	0.206
**SCYL3**	HIF1A	XPO7	PRR7	ANXA7	HEATR1	0.088
**POLG2**	CETN3	SLC35A1	CCDC92	CSK	PAN2	0.136
**NOLC1**	EBNA1BP2	TMEM109	TFDP1	NUP93	RRP1B	0.312
**POLE2**	CCNA2	GMNN	ATF1	CHEK2	POP4	0.42
**RAD51C**	PSME2	WRB	MYCBP	STMN1	OXCT1	0.106
**KIF14**	SMC4	PLK1	KIF20A	GHR	ITGAE	0.143
**SLC35A3**	RPL39L	OXCT1	SLC35A1	RB1	RPS6	0.167
**AKT1**	SMARCA4	TMEM109	RPA1	TIMELESS	AMDHD2	0.128
**MUC1**	CAPN1	THAP11	GALE	B4GAT1	GHR	0.583
**CASP2**	PGRMC1	PARP1	OXCT1	PAICS	TBP	0.069

**PARP1**	TIMELESS	ASAH1	ELAVL1	OXCT1	ARHGAP1	0.342
**FDFT1**	ELOVL6	EBP	PCMT1	BZW2	TMEM97	0.31
**CCND1**	MRPL12	SKP1	TPM1	PAICS	PFKL	0.22
**OXA1L**	PIP4K2B	SOX4	GAA	BDH1	NFKBIA	0.091
**CDC42**	MIF	PSME1	NENF	AKT1	POP4	0.153
**ADH5**	PSMD10	ICAM3	SKP1	RPA3	LYPLA1	0.107
**CYCS**	POLR2K	GTF2A2	TFDP1	DNAJC15	DYNLT3	0.112
**COPS7A**	SMARCD2	KLHDC2	CDK2	RPS6	FIS1	0.203
**GLOD4**	HN1L	SYPL1	MBNL1	HDAC2	XBP1	0.104
**DLD**	SDHB	MRPL19	ANXA7	IARS2	CPNE3	0.063
**IFRD2**	PAICS	POLR2I	NUP62	SDHB	RUVBL1	0.087
**TSPAN6**	SCP2	GTF2A2	EAPP	PSME1	SLC35A1	0.207
**CDKN1B**	ATF1	BAMBI	DDB2	TBP	LYPLA1	0.046
**PRPF4**	SDHB	TBP	STUB1	PARP2	LSM5	0.086
**CBR1**	HSD17B10	SDHB	POP4	COG4	PAICS	0.148
**NR2F6**	ERBB3	AURKB	ELOVL6	SPDEF	B4GAT1	0.099
**TSPAN4**	S100A13	LAGE3	ETFB	GAA	ENOSF1	0.353
**PSIP1**	TIMELESS	STMN1	PDS5A	MCM3	HADH	0.153
**AURKB**	CHEK1	CISD1	MYBL2	PSRC1	POLE2	0.299
**RPA3**	MYCBP	POLR2K	NRAS	MPC2	ICAM3	0.223
**HDGFRP3**	HDAC2	PAICS	NNT	SCCPDH	MBNL1	0.129
**RRS1**	RRP1B	PAICS	NUP62	PSMG1	AURKA	0.11
**EED**	USP1	CDC25A	YKT6	HDAC2	POP4	0.218
**GATA3**	NET1	XBP1	ERBB3	NUP93	SOX4	0.241
**CETN3**	TMEM50A	POLG2	DNAJC15	S100A13	HADH	0.103
**TGFB3**	OXCT1	RB1	BCL2	DDR1	SPDEF	0.13
**HIST1H2BK**	S100A13	HMG20B	GHR	CCDC92	DYNLT3	0.236
**ANXA7**	SDHB	SKP1	NRAS	CPNE3	TBP	0.37
**PUF60**	NUP88	RRP1B	COG4	BIRC5	HSD17B10	0.074
**CFLAR**	NRAS	RB1	CHEK1	AURKB	SCP2	0.177
**RPL39L**	SLC35A3	SKP1	PRR7	MYCBP	RPS6	0.152
**CHEK2**	CDCA4	ICAM3	YKT6	POLE2	TIMELESS	0.228
**HMG20B**	AKT1	CANT1	RPA1	AMDHD2	ICMT	0.076
**TPM1**	SMARCA4	EBNA1BP2	ENOSF1	TCEAL4	SCRN1	0.256
**BDH1**	MCM3	TIMELESS	B4GAT1	ICAM3	ERBB3	0.225
**LSM5**	POLR2K	CYCS	ATG3	PCMT1	MRPL19	0.117
**TXNDC9**	CRYZ	MPC2	GTPBP8	EAPP	DERA	0.098
**HN1L**	PGRMC1	PAICS	KIAA0100	NUP62	DAG1	0.223
**ZMIZ1**	ARHGAP1	SCRN1	VPS28	KAT6B	GLOD4	0.16
**PDS5A**	PCM1	NNT	SYNE2	SMC4	SCRN1	0.208
**XPO7**	PCM1	HEATR1	ATG3	HN1L	MBNL1	0.142
**COG4**	HSD17B10	CSK	ETFB	LIG1	CEBPA	0.212
**TMEM97**	CDCA4	EBP	UBE2C	FDFT1	MCM3	0.174
**TFDP1**	MYBL2	SDHB	NOLC1	PAICS	CDK2	0.313
**KAT6B**	SYNE2	EVL	ZMIZ1	BCL2	SLC37A4	0.096
**ADO**	SMARCA4	GAA	PCM1	SUV39H1	ATMIN	0.123
**JADE2**	NUP62	AURKA	FAM63A	TP53	PIP4K2B	0.357

**PCCB**	NIPSNAP1	HMG20B	PSME1	ADI1	STUB1	0.101
**KIAA1033**	ATMIN	RPS6	SLC35A1	ADI1	TLE1	0.049
**RRP1B**	SMC4	SMARCA4	NOLC1	EBNA1BP2	RPIA	0.231
**THAP11**	RUVBL1	GHR	MUC1	PCMT1	LBR	0.079
**C2CD5**	SPDEF	SLC35A1	NRIP1	GATA3	TOP2A	0.273
**POLR2I**	MIF	PAFAH1B3	ETFB	SDHB	IFRD2	0.068
**RPIA**	TMEM109	PAICS	EBNA1BP2	RUVBL1	ENOSF1	0.161
**PLCB3**	SMARCA4	CCDC92	ARHGAP1	TGFB3	AMDHD2	0.198
**ASAH1**	PRAF2	GAA	OXCT1	STAT1	PARP1	0.144
**SMARCA4**	RPA1	HSD17B10	NUP62	ENOSF1	RRP1B	0.322
**ATP5S**	SKP1	PSME1	RPA3	DECR1	ATF1	0.146
**PARP2**	USP1	NUP88	SMC4	AURKA	YKT6	0.157
**AKR7A2**	S100A13	CDK2	PRAF2	DECR1	MYCBP	0.176
**SPDEF**	ELOVL6	SLC35A1	XBP1	ARHGAP1	TCEAL4	0.204
**CCNB1**	CCNA2	AURKA	MAT2A	AURKB	PSRC1	0.197
**ERBB2**	GAA	TLE1	DDB2	ERBB3	GAPDH	0.217
**VDAC1**	EBNA1BP2	GAPDH	SDHB	MYCBP	CISD1	0.06
**GAPDH**	DAG1	TRAP1	COASY	CSK	ATG3	0.06
**CNDP2**	GAA	HN1L	TIMELESS	SMARCC1	ARHGAP1	0.16
**ADI1**	MYCBP	STMN1	ANXA7	BDH1	TFDP1	0.095
**TMEM50A**	SKP1	DECR1	TCEAL4	KLHDC2	CDC25B	0.316
**HACD3**	CISD1	AURKB	HADH	RB1	TFDP1	0.147
**YKT6**	BIRC5	UBE2C	AURKA	CCNF	TIMELESS	0.433
**BZW2**	SKP1	PRR7	TSTA3	PSME1	FDFT1	0.152
**EVL**	PAFAH1B3	AMDHD2	GAA	NNT	BCL2	0.152
**MIF**	PSME1	SKP1	BIRC5	LAGE3	MRPL12	0.278
**IARS2**	TIMELESS	ICMT	PCMT1	CDK4	WRB	0.18
**KLHDC2**	SLC35A1	ERBB3	HES1	CCDC92	BAMBI	0.372
**STUB1**	HSD17B10	AKT1	CHMP4A	PAFAH1B3	HMG20B	0.118
**ENOPH1**	SDHB	ATG3	PSMG1	PAICS	SMC4	0.101
**MRPS2**	MRPL12	TMEM109	CSK	RRP1B	NUP88	0.058
**NUP85**	TIMELESS	MCM3	TMEM109	PARP2	EZH2	0.141
**FIS1**	TSPAN6	HMG20B	EML3	KLHDC2	COPS7A	0.111
**NUSAP1**	TOP2A	CDK1	DERA	CDK2	CDC45	0.219
**MRPS16**	ICAM3	LAGE3	CDK4	PSME1	ATP5S	0.224
**NPDC1**	GAA	TSTA3	CTSD	EVL	EML3	0.212
**DERA**	GMNN	CDK2	NUSAP1	CDC45	MPC2	0.104
**UBR7**	PCMT1	SMC4	USP1	CDCA4	CCP110	0.109
**ISOC1**	MPC2	GHR	TIMELESS	ELOVL6	IARS2	0.202
**CCDC92**	GAA	RRAGA	PLCB3	HES1	GHR	0.163
**SCAND1**	DDB2	TCEAL4	HSPB1	PSME1	CIRBP	0.169
**ZDHHC6**	PAICS	EZH2	TFDP1	PCMT1	PARP2	0.186
**TIMM9**	ATF1	POLE2	NUP62	XBP1	TSEN2	0.122
**GMNN**	POLE2	BIRC5	GTF2A2	RPA3	AURKB	0.348
**CDCA4**	USP1	CHEK2	CCNF	LAGE3	ICAM3	0.331
**NENF**	PSME1	TSTA3	TSEN2	CDC42	OXCT1	0.292
**DNAJC15**	CRYZ	GTF2A2	RPA3	CETN3	POLR2K	0.2

**HEBP1**	SPDEF	GHR	CIRBP	ARHGAP1	TCEAL4	0.111
**CD320**	EBNA1BP2	SDHB	SMARCA4	HSD17B10	PGRMC1	0.084
**CHMP4A**	TPM1	SMARCA4	PCMT1	TCEAL4	CISD1	0.14
**HEATR1**	XPO7	SMARCC1	SCYL3	NOLC1	SOX4	0.171
**CISD1**	CCNH	AURKB	BDH1	RB1	SLC35A3	0.288
**SUV39H1**	AURKA	CDC25A	YKT6	BRCA1	ADO	0.197
**VPS28**	ENOSF1	SLC35A3	SMARCC1	ZMIZ1	SYNE2	0.114
**KIF20A**	CCNA2	AURKA	BIRC5	SMC4	TOP2A	0.244
**METRN**	AKT1	PRR7	MYCBP	MELK	NUDCD3	0.092
**LAGE3**	ICAM3	PAFAH1B3	MYCBP	MRPS16	S100A13	0.242
**PXMP2**	PSME1	S100A13	MYCBP	OXCT1	TIMELESS	0.102
**AMDHD2**	GAA	PAFAH1B3	GALE	HSPB1	CCDC92	0.2
**FBXO11**	CASP2	CHEK1	HADH	RHOA	MCM3	0.051
**MTERF3**	POLE2	TSEN2	CCNA2	SLC35A1	POLR2K	0.141
**PSMD10**	SKP1	NRAS	HDAC2	RHOA	ADH5	0.218
**TSEN2**	TMEM109	MRPL19	CDC25A	MAT2A	MRPL12	0.119
**PRR7**	TSTA3	NUDCD3	ENOSF1	SDHB	CISD1	0.151
**GTPBP8**	CRYZ	TIMM9	SLC35A1	CYCS	TXNDC9	0.065
**DENND2D**	DDB2	INPP4B	DECR1	SCRN1	SYPL1	0.152
**ARPP19**	NRAS	CEBPA	RPS6	ATF1	CBR1	0.109
**ATG3**	CCNH	EBNA1BP2	EZH2	POLR2K	ENOPH1	0.093
**WDR61**	MYCBP	ATP5S	RPA3	POP4	CRYZ	0.27
**CANT1**	KIF20A	HMG20B	TLE1	MRPS2	NUP62	0.088
**FAM63A**	NNT	DDB2	GATA3	DDR1	TCEAL4	0.217
**ATF1**	NRAS	SLC35A1	POLE2	TBP	USP1	0.191
**TSTA3**	ETFB	GAA	MYCBP	TRAP1	COG4	0.153

[1] *University of Manchester, Department of Computer Science, Manchester, UK.*
[2] *Taif University, Department of Computer Science, Taif, Saudi Arabia.*
[3] *Department of Chemical Engineering and Biotechnology, University of Cambridge.*
[4] *Department of Biology and Biological Engineering Chalmers University of Technology, Sweden.*
[5] *Alan Turing Institute, NW1 2DB, London, UK.*
